# Social Structure Facilitated the Evolution of Care-giving as a Strategy for Disease Control in the Human Lineage

**DOI:** 10.1038/s41598-018-31568-2

**Published:** 2018-09-27

**Authors:** Sharon E. Kessler, Tyler R. Bonnell, Joanna M. Setchell, Colin A. Chapman

**Affiliations:** 1 Department of Anthropology, Durham University, South Rd, Durham, DH1 3LE UK; 2 Department of Psychology, University of Lethbridge, Lethbridge, Alberta Canada; 3 Department of Anthropology, McGill University, Montréal, H3A 2A7 Québec Canada; 4 School of Life Sciences, University of KwaZulu-Natal, Pietermaritzburg Scottsville, South Africa; 5 Shaanxi Key Laboratory for Animal Conservation, Northwest University, Xi’an, China

## Abstract

Humans are the only species to have evolved cooperative care-giving as a strategy for disease control. A synthesis of evidence from the fossil record, paleogenomics, human ecology, and disease transmission models, suggests that care-giving for the diseased evolved as part of the unique suite of cognitive and socio-cultural specializations that are attributed to the genus *Homo*. Here we demonstrate that the evolution of hominin social structure enabled the evolution of care-giving for the diseased. Using agent-based modeling, we simulate the evolution of care-giving in hominin networks derived from a basal primate social system and the three leading hypotheses of ancestral human social organization, each of which would have had to deal with the elevated disease spread associated with care-giving. We show that (1) care-giving is an evolutionarily stable strategy in kin-based cooperatively breeding groups, (2) care-giving can become established in small, low density groups, similar to communities that existed *before* the increases in community size and density that are associated with the advent of agriculture in the Neolithic, and (3) once established, care-giving became a successful method of disease control across social systems, even as community sizes and densities increased. We conclude that care-giving enabled hominins to suppress disease spread as social complexity, and thus socially-transmitted disease risk, increased.

## Introduction

The evolution of care-giving in the human lineage provided our ancestors with an unprecedented ability to modify the progression of diseases through their populations^1^ and likely had far reaching implications for the evolution of both pathogens and the human immune system. Care-giving would have dramatically altered disease transmission patterns by increasing contacts between infected and susceptible individuals, thus selecting for pathogens that would transmit effectively through these new contact patterns and for a host immune system that is specialized for care-giving. That we are the only species to have evolved widespread, cooperative care-giving for ill individuals suggests that it evolved as a response to conditions that only occurred in the human lineage.

Five decades of research on our closest living relatives, the other apes, have revealed that the human lineage evolved a unique suite of potentially correlated cognitive and social traits^2–7^. After hominins diverged from the rest of the primate order, brain size increased in the human lineage and ancestral capacities for social learning led to greater cumulative culture and increasingly complex technology^8,9^. A shift to cooperative breeding^4,5,10–14^ increased the complexity of social networks and is thought to have selected for psychological changes, including greater empathy^3,15^, the ability to understand the mental states of others, and hyper-cooperation^6,7^.

Current research^16^ suggests that this package of cognitive traits may have been key to enabling hominins to recognize when other individuals were sick and in need of care. Detecting disease cues (disease recognition) and processing social information^7,8,17,18^ use the same neural networks, i.e., face- and odor-perception networks^16^. When we look at someone’s face or smell their body odors, we glean information about their identity, emotions, and motivations, and simultaneously obtain information about the person’s health status (changes in facial coloration due to fever or rashes, odor changes due to sweating from a fever). The same is likely to be true for voice processing; changes in someone’s voice can alert the listener to respiratory infections. These neural overlaps in disease recognition and social cognition provide compelling evidence that the two evolved together. However, this work also shows that disease recognition must be recognized as cognitively complex independent of social cognition^16^; in addition to integrating multisensory cues from the respective sensory networks, it involves “superadditive” processing^16^. This superadditive processing uses cognitive pathways that are not involved when evaluating cues from healthy individuals nor when the visual or the olfactory cues for disease were evaluated individually, providing clear evidence that disease recognition requires complex, multi-level cognitive processing^16^. For these reasons, we suggest that disease recognition is a previously unrecognized, and crucial, element of the social and cognitive traits that are associated with increasing social complexity in the genus *Homo*^3,6,7,18–20^.

Cross species studies of nonhuman animals indicate that increased sociality brings intense selection to limit the costs of disease exposure^21–23^, suggesting that the same would have been true of hominins. Recent work in paleogenetics, pathogen genomics, and evolutionary medicine has shown that not only have hominins been infested with numerous pathogens since before the advent of agriculture and animal domestication^24,25^, but also that their immune defenses were under heavy selection as hominins expanded into new environments, encountered new animal communities^26^, and hybridized across hominin species acquiring genetic variation at immune loci^27–33^. Theoretical and modeling studies indicate that even low levels of relatively ineffective care-giving could have enabled hominins to limit the progression of disease through their populations and reduce the severity of outbreaks^1,34,35^.

The origins of care-giving are difficult to pinpoint in the fossil record because, although numerous hominin specimens from *Homo erectus* to *H*. *sapiens* bear evidence of recovering from severe illnesses and injuries^36–38^, nonhuman animals survive similar afflictions without care^39,40^, and care-giving leaves no other fossilized evidence. This means that survival cannot be taken as proof that sick and injured hominins received care. However, regardless of when care-giving evolved, the most parsimonious explanation is that it evolved through pre-existing networks, such as kin networks. Kinship underpins much of primate sociality^41^ and is the backbone of the networks through which primates care for immatures. Moreover, theoretical and modeling evidence indicates that the inclusive fitness benefits of caring for ill kin mitigates some of the costs of care-giving, making it an evolutionarily stable strategy^1,34,35^.

The evolution of human social organization, and thus the structure of kin networks, could have either supported or hindered the evolution of care-giving. Here, we simulate the evolution of care-giving for the diseased in a basal primate social system, in which the sole care-giving bonds occur between mother and infant (hereafter *basal primate*), and compare this with feasibility of the evolution of care-giving in the three leading hypotheses of ancestral human social structure: pair-bonded (*pair-bonded*)^42,43^, pair-bonded with indirect reciprocal altruism from a random individual (*indirect reciprocity*)^44–46^, and cooperative breeding with assistance from kin (*kin-based*)^12,14,47,48^. We show that kin-based cooperative breeding, in contrast to the other social systems, likely facilitated the evolution of care-giving in the human lineage, but once effective care was established, care-giving networks would have become more flexible, potentially contributing to the diversity and flexibility of human social systems.

## Results and Discussion

We created two models (the Ineffective Care Model and the Effective Care Model) and ran each with each of four social systems (basal primate, pair-bonded, indirect reciprocity, and kin-based, see Table S1. Model code and detailed descriptions are in the Supplementary Files and Text). We provide a brief summary of the two models, the four social systems, then the details of the models.

The Ineffective Care Model simulated the difficulties of establishing care-giving as a method of disease control. It subjected communities of ineffective care-givers to repeated introductions of novel diseases. Each time care was given, the reduction in the recipient’s pathogen replication rate was reduced by a random number less than or equal to the values taken from the literature for that type of care (Table 1). Thus, the benefits of ineffective care both vary randomly and are smaller than the benefits reported in the literature. Novel disease were generated by setting the disease parameters (death rate, transmission rate, and recovery rate) to random numbers within the range expected to maximize care-giving in all social systems (≤0.5)^1^. As the disease parameters influence how the disease spreads through the population, this creates unique dynamics for each disease. After a disease goes extinct, a novel disease is created with new, randomly selected disease parameters and introduced in the next time step.

**Table 1:** Parameters used in the Ineffective Care Model and the Effective Care Model.

Parameter	Ineffective Care Model	Effective Care Model	Citation/Justification
Transmission rate	</=0.5	0.1–0.9	Explores range of values
Death rate	</=0.5	0.1–0.9	Explores range of values
Recovery rate	</=0.5	0.1–0.9	Explores range of values
Carrying Capacity	50, 100, 150, 200	50, 100, 150, 200	^1, 49^
Landscape	200 km^2^	200 km^2^	^1, 49^
Initial prevalence	5, 25, 50,75%	5, 25, 50,75%	Explores range of values
Initial Population	20	20	Enables network structure to develop
Pathogen load	1000	1000	Value is arbitrary. Importance is change over time.
Standard deviation of intelligence distribution	0.15	0.15	^78^
Kin Recognition	0.125–0.5	0.125–0.5	^79^
Food benefit	</=0.25	0.25	^80^, for higher rates^81,82^:
Water benefit	</=0.2	0.2	^83, 84^
Hygiene benefit	</=0.5	0.5	^85– 87^
Protection benefit	</=0.2	0.2	No existing data was found. Value set to the lowest benefit of the other three benefit types.
Movement radius	10 grid cells	10 grid cells	^49, 53^
Infection radius	10 grid cells	10 grid cells	^49, 53^

The Effective Care Model differed from the Ineffective Care Model by simulating populations in which effective care has been established and systematically varying the disease parameters. Under this model, the benefits of care were fixed at the values found in the literature (Table 1). Disease parameters were varied simultaneously between 0.1 and 0.9 (10%–90%). For example a disease with a severity of 0.1 had a death rate of 0.1, transmission rate of 0.1, and recovery rate of 0.9. When one disease goes extinct, the same disease (with the same disease parameters) is introduced in the next time step. This enabled us to systematically examine the effects of disease parameters over time.

Both models were run with each of four social systems (basal, pair-bonded, indirect reciprocity, and kin-based). In the basal primate social system, care was given only from mothers to offspring. The pair-bonded social system^42,43^ included parent to offspring care and obligate care between pair-partners. The indirect reciprocity social systems added obligate care from a randomly selected altruist to the pair-bonded care system^44–46^. The kin-based social systems included basal mother-offspring care and care from siblings, grandparents, aunts/uncles, nieces/nephews, and cousins^14,47^.

The models simulate a hominin community with fission-fusion dynamics in which subsets of individuals are out of contact with other subsets^1,49^. Community size is limited by four carrying capacity settings: 50, 100, 150, and 200 individuals. This covers reconstructed^50^ and actual^51,52^ community sizes for chimpanzees. Moreover, the range of 50–100 individuals covers the range of median community sizes reconstructed for early *Homo*, *H*. *habilis*, *H*. *erectus*, *H*. *heidelbergensis*, *H*. *neandertalensis*, and *H*. *sapiens*^1,49^. The larger community sizes (150, 200) are greater than those predicted for ancestral hominins and are included to investigate the robusticity of care-giving as community sizes increased in the human lineage^24^. These values encompass the lower range of modern hunter-gather communities (125–200 individuals), including populations on four continents and living in habitats ranging from tropical forest to the Arctic coast^53^. (We didn’t conduct simulations of larger community sizes, because the simulations that we currently present already suggest that care-giving evolved first in smaller, not larger groups).

With a range of 200 km, our carrying capacities produce densities of approximately 0.25, 0.5, 0.75, and 1 individual/km^2^, thus approximating the density ranges and confidence intervals predicted for the above *Homo* species^1,49^. The initial prevalence of the diseases in the population varied (5%, 25%, 50%, and 75%). All possible parameter combinations were run 100 times for each social system.

In all models the first generation of hominins was assigned heritable intelligence scores. Intelligence is conceptualized as a general ability for efficient information processing^54^, with more specific cognitive abilities like disease recognition and social learning being aspects of intelligence. When an agent reproduces, the offspring’s intelligence is drawn from a normal distribution with the mother’s intelligence as the mean and a standard deviation of 0.15 (see below for social system specific differences). This represents the 15% standard deviation in the human IQ scale (Table 1). Thus, intelligence of the population changes overtime depending upon whether high or low intelligence individuals are reproducing more rapidly. The ability to recognize when another individual is infected (*disease recognition*) is an aspect of intelligence and a prerequisite for providing care. Disease recognition does *not* require that individuals have an abstract concept of disease because it functions on the ultimate level of causation (similarly to how kin recognition operates without individuals requiring an abstract concept of kin).

All carers, except for pair-partners and altruists, cease providing care when the costs exceed the benefits, according to a modified version of Hamilton’s rule^55^ in which the costs and benefits are expressed in terms of disease risk (infectiousness of the infected individual, death rate of the disease, and recovery rate). When an individual receives care, this reduces that individual’s pathogen replication rate according to an exponential dose-response curve^56,57^, reducing the individual’s infectiousness, lowering the probability of dying, and increasing the likelihood of recovery. Exponential dose-response curves are used to estimate disease risks in numerous bacterial, protozoal, and viral infections^56,57^. They require fewer assumptions than other curves, making them appropriate for modeling non-specific diseases^56,57^. When multiple individuals provide care to the same ill individual (*cooperative care*), carers can learn additional care-giving skills, with learning being a function of the carer’s intelligence. There are four possible types of care: providing food, water, assistance with hygiene, and protection, with respective reductions in pathogen replication rates based on values from the literature (Table 1). Each sick individual can only receive each type of care once per infection, but individuals do not need to receive all types of care to recover. If a sick individual does not receive all types of care in one time step and remains infected in subsequent time steps, it may receive additional forms of care if its care-giving network expands through population growth, if its carers learn new skills, or if carers who previously failed to recognize the disease later do.

**Figure 1:**
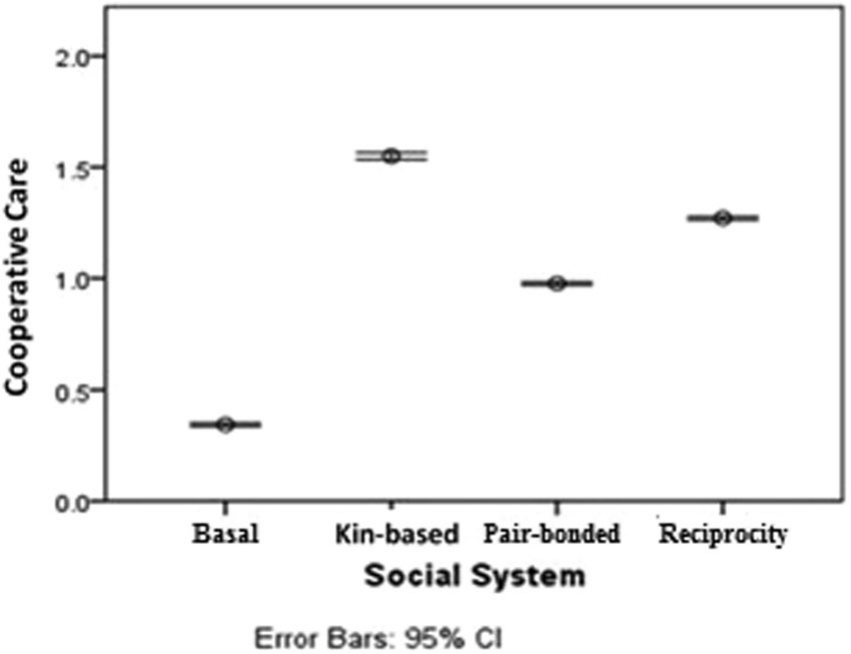
Mean levels of cooperative care produced by each of the four social systems under the Ineffective Care Model. Each diseased individual could receive up to four types of care. This graph includes all community sizes and initial disease prevalence settings.

Correspondence between the care-giving rates produced by our models and data from hunter-gather communities suggest that our models have high ecological validity. Data from hunter-gather societies (Ache and Hiwi) shows that on average, breeding pairs with dependents received assistance from 1.3 helpers, including provisions from 0.8 males^46^. Under the Ineffective Care Model, diseased individuals in the pair-bonded, indirect reciprocity and kin-based systems received care from approximately 1, 1.3 and 1.5 carers (Fig. 1) with slightly higher rates under the Effective Care Model (Fig. S3). Similarly Ache and Hazda individuals, on average, exchange food during injury/illness with 2–3% of the other community members in a given year^58^. Under the Ineffective Care Model, diseased individuals living in the smallest communities have care-giving relationships with approximately ≤2% (pair-bonded), ≤2.6% (indirect reciprocity), and ≤3% (kin-based) of the community (calculated as the number of carers/carrying capacity of 50 individuals). Though the percentage of dyads with care-giving relationships decreases as community size increases, it also increases under the Effective Care Model (Fig. S3). Overall, our data shows a remarkable similarity to the care-giving patterns reported for hunter-gathers, indicating that the ecological validity of our models is high.

Our results from the Ineffective Care Model demonstrate that the ancestral human social system had a dramatic effect on the feasibility of the evolution of care-giving for the diseased. Social system accounted for most of the variation in cooperative care-giving (88%), even more of the variation in the percentage of infected agents who received care (92%), and a very large proportion of the variance in the mean number of care-giving skills per agent (77%) (Table 2). The effects of social system were much larger than the average effects of community size and initial disease prevalence; although both varied greatly (carrying capacity range: 50–200; initial prevalence range: 5–75%), they accounted for <5% of the variation (r2m in Table 2).

**Table 2:** Estimated effect sizes (Effect) and standard errors (STE) for the Ineffective Care and Effective Care models.

Ineffective Care Model	Cooperative Care^a^		Percent Care^b^		Skills^c^		Δ Intelligence^d^		Extinction^e^		Spread^f^	
	Effect	STE	Effect	STE	Effect	STE	Effect	STE	Effect	STE	Effect	STE
Intercept	1.035	0.259	36.728	13.998	1.361	0.167	−0.057	0.020	33.843	1.890	5.563	0.900
CS	0.104	0.028	0.861	0.273	−0.005	0.006	−0.035	0.004	31.321	1.917	3.947	0.446
IP	−0.051	0.035	−5.182	1.421	−0.062	0.026	0.009	0.003	3.636	1.403	1.774	0.472
CS* IP			0.795	0.080	0.027	0.002			2.770	1.225	0.971	0.067
R2m	0.042		0.033		0.031		0.041		0.095		0.370	
R2c	0.922		0.953		0.798		0.095		0.097		0.461	
Social System	0.88		0.92		0.767		0.054		0.002		0.091	
**Effective Care Model**	**Cooperative Care** ^**a**^		**Percent Care** ^**b**^		**Skills** ^**c**^		**Δ Intelligence** ^**d**^		**Extinction** ^**e**^		**Spread** ^**f**^	
	**Effect**	**STE**	**Effect**	**STE**	**Effect**	**STE**	**Effect**	**STE**	**Effect**	**STE**	**Effect**	**STE**
Intercept	1.070	0.307	35.960	13.295	1.404	0.185	−0.007	0.018	4.072	0.152	5.066	0.898
CS	0.088	0.024	1.348	0.694	0.018	0.007	−0.002	0.002	1.004	0.117	2.578	0.353
DS	−0.332	0.132	−14.318	3.918	−0.351	0.152	−0.099	0.038	0.718	0.223	1.246	0.536
IP	−0.043	0.028	−4.582	1.267	−0.078	0.029	0.024	0.007	0.772	0.014	1.908	0.480
CS*DS	0.004	0.001	0.786	0.031	0.003	0.001	−0.005	0.001	0.197	0.007	0.567	0.010
CS*IP	−0.011	0.001	0.700	0.031	0.011	0.001	0.002	0.001	−0.092	0.007	0.711	0.010
DS*IP	0.043	0.001	0.501	0.032	0.026	0.001	0.012	0.001	0.230	0.007	0.249	0.010
CS*DS*IP	−0.018	0.001	0.031	0.032	0.011	0.001	−0.001	0.001	−0.152	0.007	−0.142	0.010
R2m	0.196		0.254		0.307		0.254		0.486		0.577	
R2c	0.946		0.421		0.890		0.421		0.564		0.822	
Social System	0.75		0.167		0.583		0.167		0.078		0.245	

^a^Cooperative care is the mean number of care events received by diseased individuals. ^b^Percent Care is the percentage of diseased individuals who received care from at least one carer. ^c^Skills is the mean number of care-giving skills per agent in the population. It represents the social learning of care-giving skills because agents with more than one skill have acquired the addition skills through social learning. ^d^Δ Intelligence is the change in population intelligence. ^e^Extinction is the time to disease extinction. ^f^Spread is the percentage of infected who were infected by another agent. Both the Ineffective Care and the Effective Care models account for changes in community size (CS) and the initial prevalence of the diseases (IP), while the Effective Care model also accounts for disease severity (DS). R2m (marginal)^77^ is the percentage of the variation explained by the fixed effects (Ineffective Care Model: Community Size, Initial Prevalence, Community Size * Initial Prevalence; Effective Care Model: Community Size, Initial Prevalence, Disease Severity, and interaction effects), whereas R2c (conditional) is the percentage of the variation explained by taking into account both the fixed and random portions (Social System) of the model. Social system is the percent of the variation explained by the effect of social system and is calculated as the difference between R2m and R2c.

**Figure 2:**
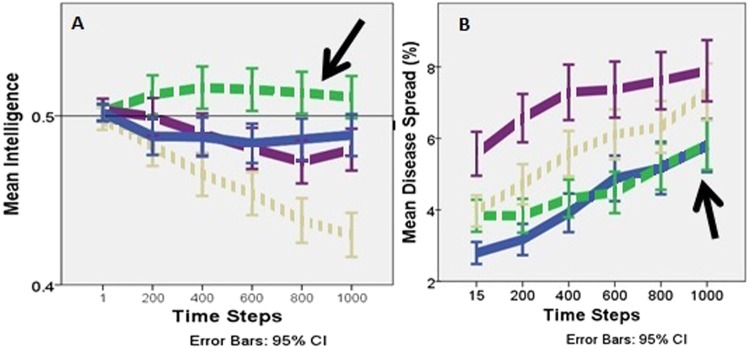
Change over time in the small communities’ mean population intelligence **(A)** and disease spread **(B)**. The arrows highlight that only the kin-based communities (green medium dashes) show an increase in mean population intelligence over time **(A)** and disease spread that approximates the spread in the basal primate communities (solid blue line) **(B)**. The reference line at 0.5 **(A)** highlights the average intelligence of the populations at the start of the model runs. In the model, disease recognition is a function of intelligence, thus an increase in intelligence reflects selection for disease recognition and care-giving. The kin-based communities are shown with a medium dashed line, basal primate in a solid blue line, pair-bonded in a short grey dashed line, and indirect reciprocity in a long purple dashed line.

Disease recognition (a prerequisite of care-giving) and social learning of additional care-giving skills are functions of intelligence, so increasing intelligence indicates positive selection for care-giving. This occurred only in the small, kin-based communities (Fig. 2). The decreases in intelligence seen in all other social systems indicate selection against care-giving (Fig. S1). Small kin-based communities produced positive selection for care-giving because care-giving under those conditions suppressed disease spread. Figure 2 shows that the 95% confidence intervals for disease spread in the small, kin-based system overlap with the confidence intervals for the basal primate system. This contrasts with the pair-bonded and indirect-reciprocity curves where disease spread is higher than in the basal system. In the large communities, all social systems show increased disease spread relative to the basal system (Fig. S1). This suggests that as the ancestral human social system evolved from the basal primate system to small communities of kin-based cooperative breeders, care-giving limited diseases from spreading beyond the spread occurring in the basal primate system.

Even though care-giving increases contacts between susceptible carers and infectious individuals, small, kin-based communities had two advantages that made care-giving an effective method of disease control: highly cooperative care and network modularity. Infected individuals received the most care under the kin-based system (Fig. 1), meaning that the cumulative benefit to the infected individual was greater. Carers were also less vulnerable to infection because they shared the costs of care-giving with more carers. Indirect reciprocity provided care to the highest percentage of diseased individuals (Fig. S2), but also had the highest disease spread (Fig. 2). Because altruists provided care regardless of kinship or the risks, they reduced network modularity and increased spread. In contrast, while pair-bonded agents always provided care to their pair partner, the care remained within the kin network. This limited spread relative to the indirect reciprocity networks, but still produced greater spread than in the basal primate system. Thus modularity of the kin networks combined with highly cooperative care, enabled care-giving to become established in the small, kin-based communities as a method of disease control. The modular nature of kin-based care in our models is supported by empirical evidence from the BaYaka foragers. The BaYaka share plant knowledge for medicinal purposes within kin networks, while plant knowledge for foraging and social beliefs is shared more widely among the camp^59^.

That care-giving was an effective method of disease control when community sizes were small and population densities were low suggests that care-giving evolved before the large, high density communities associated with the advent of agriculture in the Neolithic Age^24^. The community sizes (~50–100) span the range predicted for the genus *Homo* and the densities (0.25–0.5 individuals/km^2^) are generally in the lower ranges predicted for these species^1,49^. This indicates that kin-based cooperative breeding networks in small, low density hominin communities may have facilitated evolution of care-giving long before the beginning of agriculture in the Neolithic^24^.

While the Ineffective Care Model simulated the establishment of care-giving in populations which provide ineffective care during repeated introductions of novel diseases, the Effective Care Model simulated populations that give effective care and explored the effects of varying disease severity on the flexibility of the care-giving networks. The reduction in pathogen replication rate produced by each type of care was fixed at the values obtained from the literature (Table 1). The three disease parameters were varied simultaneously between 0.1 and 0.9 in increments of 0.1 (referred to as *disease severity* in the Effective Care Model). As before, we varied community sizes (small: capped at 50 or 100; large: capped at 150 or 200) and the initial prevalence of the disease (5%, 25%, 50%, and 75%). All parameter combinations were run 100 times for each social system.

**Figure 3:**
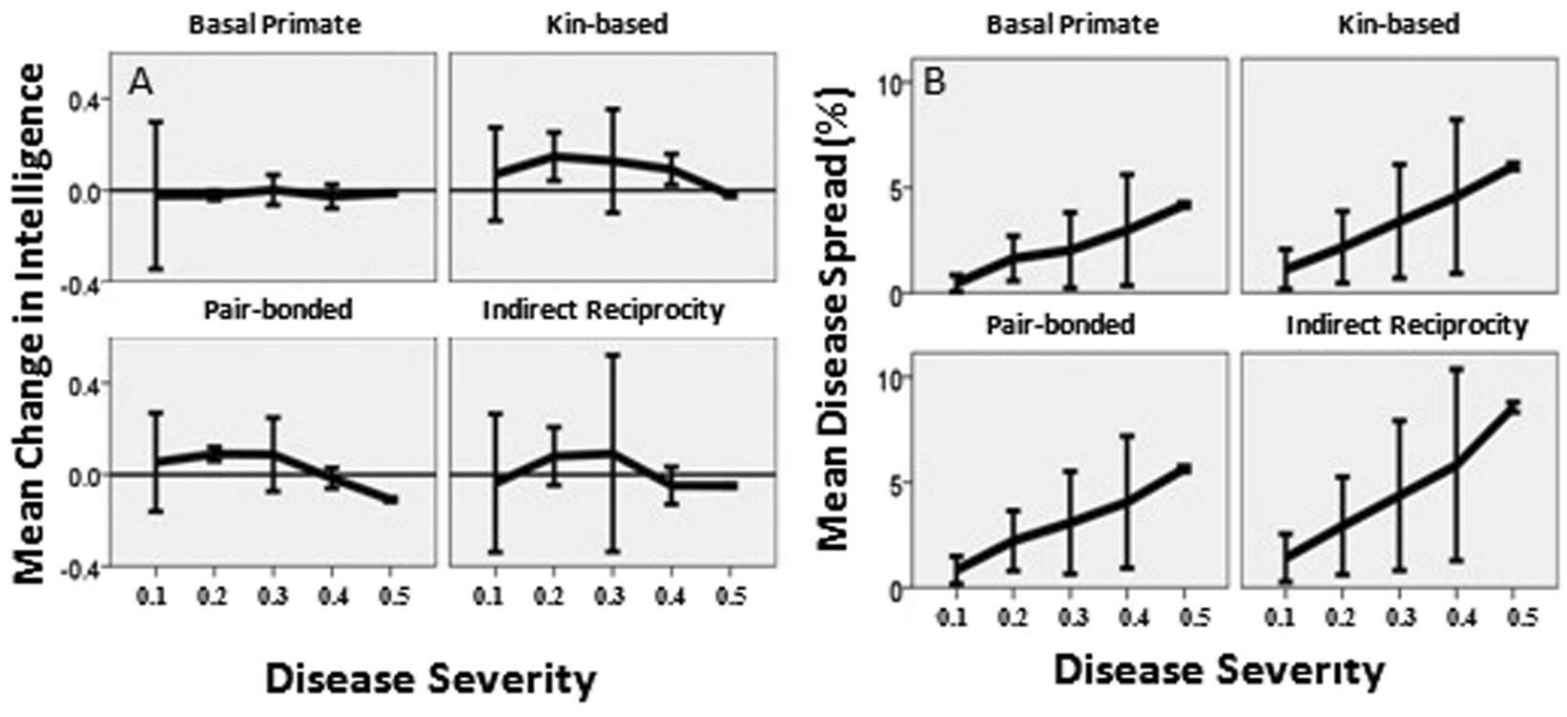
Net change in intelligence **(A)** and disease spread **(B)** in each of the four social systems in response to diseases with severities ranging from 0.1 to 0.5. The basal primate social system produces very little change in intelligence. A: While the pair-bonded, indirect reciprocity, and kin-based systems each produced increases in intelligence at disease severities of 0.3 or below, only the kin-based system increased intelligence in response to diseases with severities of 0.4. B: The highly overlapping confidence intervals indicate that levels of disease spread are similar across social systems under the Effective Care Model. In both graphs, the error bars are 95% confidence intervals. The graph pools across community sizes and initial prevalences.

The Effective Care Model showed that once effective care is established (benefits fixed at rates in the literature, Table 1), care-giving networks become more flexible in that care-giving is a successful strategy of disease control in pair-bonded, indirect reciprocity, and kin-based networks. Care-giving in the pair-bonded, indirect reciprocity, and kin-based social systems each suppressed disease spread to levels similar to the basal primate system, as shown by the highly overlapping confidence intervals produced by mild diseases across social systems (Fig. 3). Moreover, each of the three social systems with cooperative care produced positive selection for intelligence (reflecting selection for disease recognition, social learning of care-giving skills, and care) in response to diseases with severities of 0.1–0.3 (Fig. 3). However, only the kin-based system maintained positive selection in response to diseases through a severity of 0.4, suggesting that the higher levels of cooperative care in the kin-based social system (Fig. S3) made care-giving in these communities effective with more severe diseases. In the basal primate social system, where care is only given by the mother to the offspring, there is very little selection for disease recognition and thus very little evolution of intelligence (Fig. 3). Overall, the results indicate that once effective care was established in hominin populations, care-giving networks became increasingly flexible, potentially contributing to the evolution of extreme social flexibility in humans.

**Figure 4:**
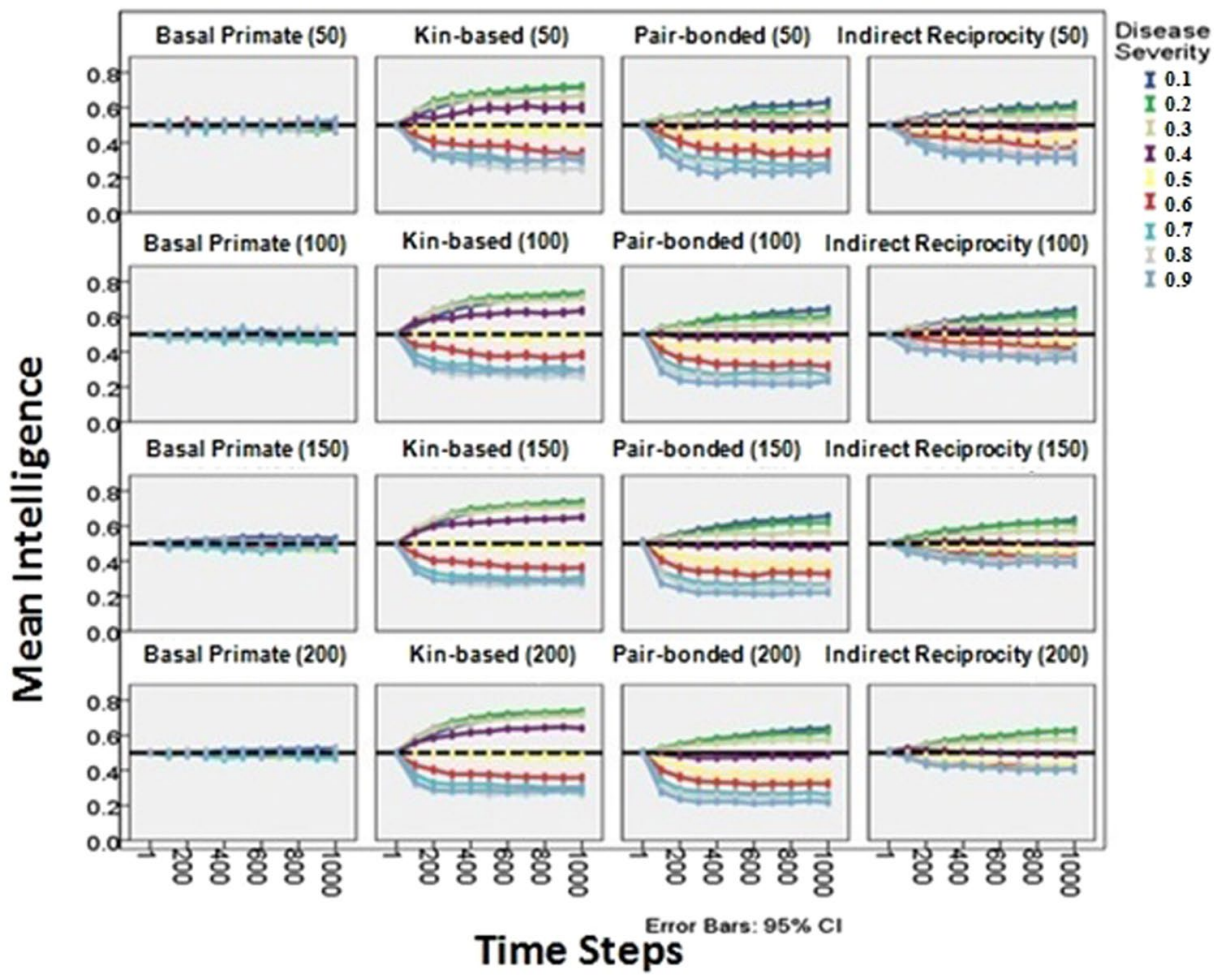
Change in intelligence over time as produced by diseases varying in severity from 0.1–0.9 (colors), under the four social systems (columns), and at the four different community sizes (rows, in parentheses). Across social systems the least severe diseases produced the greatest increases in intelligence and the most severe produced the greatest decreases. Community size did not have a large effect. Error bars are 95% confidence intervals. The graph pools across initial prevalences.

Over the course of hominin evolution, the greatest selection for increasing intelligence and care-giving would have been produced by high prevalence, low severity diseases. Across the pair-bonded, indirect reciprocity, and kin-based social systems, milder diseases generally produced greater increases in intelligence, reflecting stronger selection for disease recognition, social learning of care-giving, and care. More severe diseases typically produced greater decreases in intelligence, reflecting stronger selection against disease recognition, social learning of care-giving, and care. Figure 4 shows that the negative relationship between disease severity and intelligence is constant across social systems when community size is held constant (rows) and within a social system across community sizes (columns). The colors represent varying disease severities and the order of the colors is largely constant across the panels. Comparisons of the effect sizes across care-giving response variables show that disease severity had a negative effect on cooperative care, the percentage of diseased who received care, and mean number of socially learned care-giving skills (Table 2).

**Figure 5:**
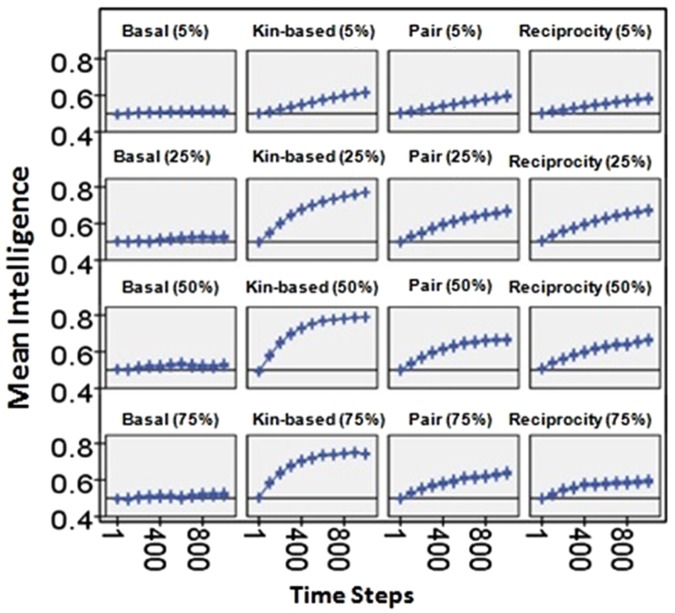
Change in intelligence overtime as produced by disease with a severity of 0.1 under four social systems (columns) and the four initial prevalences (rows, in parentheses). Across social systems the basal social system produced less change than the others. Increasing the initial prevalence from 5% to 50% increases the slope of the change in intelligence. This occurs because the disease affects more individuals, more of the population is engaged in care-giving, and selection occurs more rapidly. Error bars are 95% confidence intervals. The graph pools across community sizes.

Initial disease prevalence changed the slope of the curves, with higher initial prevalence (up to 50%) producing more positive slopes (columns in Fig. 5). Higher prevalence diseases elicit greater levels of caring and faster population turn-over, generating faster evolution. At an initial prevalence of 75%, care-giving was reduced because only healthy agents gave care. These results indicate that once effective care was established, mild, high prevalence diseases would have selected for increasing intelligence and care-giving in pair-bonded, indirect reciprocity, or kin-based social systems. Additionally, very severe diseases which would have selected against care-giving may have been relatively rare, been self-limiting in low density host populations due to high mortality^60^, and/or prompted hominins to use other strategies for disease control (i.e., avoidance^1,16^). Thus, over the course of human evolution, frequent, high prevalence, mild diseases may have had a more sustained effect on care-giving behavior than rare, self-limiting, severe diseases. While it is difficult to reconstruct the virulence of ancient pathogens, several diseases which have persisted in human populations since before the Neolithic^24^ have similar mortality today to those which produced positive selection in the models (i.e., approximately ≤40% death rate in untreated populations), including whooping cough (≤4% in infants)^61^, typhoid (12–30%)^62^, and tuberculosis (≤45%)^63^.

The Effective Care Model shows that with effective care-giving, disease recognition remains under positive selection even when population densities increase to the upper limits of those reconstructed for ancestral hominin species (1individual/km^2^) and community sizes typically observed in humans (>100 individuals^53^). Although larger communities needed longer to eliminate diseases and experienced greater disease spread (positive effect sizes of community size in Table 2), community size had very little effect on the evolution of disease recognition, social learning of care-giving skills, and intelligence (columns, Fig. 4). This suggests that, although care-giving likely evolved in small, low-density, kin-based communities under conditions similar to the Ineffective Care Model, once effective care-giving was established, it remained under positive selection as a mechanism of disease control, even as community sizes and densities increased with the advent of farming and animal domestication in the Neolithic Age^24^.

The positive feedback loop produced by the correlated evolution of disease recognition and social learning as elements of general intelligence^54^, is consistent with the idea that care-giving evolved as part of a suite of cognitive and emotional adaptations (enhanced empathy, theory of mind, hyper-cooperation, etc.)^3,7^ that are associated with the genus *Homo*, the shift to cooperative breeding^4,6,11,13^ and caring for altricial infants^64^. Our results support kin selection^55^ over reciprocal altruism^44^ as the driving selection pressure behind care-giving. The kin-based cooperative breeding social system included care from parents, siblings, grandparents, aunts/uncles, nieces/nephews, and cousins^14,47^ and allowed individuals to cease care-giving when the costs exceeded the fitness benefits specific to the kinship relationship. In contrast, the pair-bonded^42,43^ and indirect reciprocity systems^44–46^ included, in addition to the basal mother-offspring care and father-offspring care, obligate care between pair-partners and (in the indirect reciprocity system) altruists. The lower levels of cooperative care in the pair-bonded and reciprocity social systems caused the carers to provide risky care without the extended care-giving networks present in the kin-based cooperative breeding social system, thus increasing disease spread beyond the control of care-giving. However, our findings should *not* be viewed as mutually exclusive with existing hypotheses for the evolution of care-giving (i.e., provisioning by nonkin via indirect reciprocity networks^44–46^); the Effective Care Model indicates that once care-giving was established and individuals provided effective care, pair-bonded networks and reciprocity-based networks would also produce positive selection for disease recognition and care during mild illnesses. This suggests that the indirect reciprocity networks reported in several hunter-gather studies, in which nonkin provision diseased community members in exchange for similar assistance from the community in the future^44–46^, evolved secondarily to care-giving along kin networks.

Moreover, care for the injured^44–46^ should not be viewed as mutually exclusive with care for the diseased. Ancestral hominins probably did not have an understanding of Germ Theory or infectiousness, so providing care for non-infectious individuals (including the injured^44–46^) would have enabled care-giving to occur in situations with zero risk of transmission, likely promoting the practice of care-giving more broadly.

Arguments for human uniqueness have often invoked the cognitive, social, and emotional adaptations that are attributed to the evolution of cooperative breeding in the genus *Homo*^4,6,11,20^. Our findings suggest that the combination of these psychological specializations with kin-based cooperatively breeding networks may have provided the scaffolding for the evolution of care-giving for the diseased. Because behavioral responses to disease influence disease transmission patterns, they co-evolve with immune responses^34,35^. Symptom expression is a result of complex interactions between pathogens and the host immune system^34,35^. It is intriguing that humans, in contrast to other primates, have lost their body fur^65^ and have white sclera^66^ around their eyes, making sores, rashes, fevers, and bloodshot eyes more visible. This may indicate selection on immune responses to display and exaggerate, rather than conceal, symptoms as a mechanism for soliciting care in care-giving contexts^34,35^. It opens the intriguing possibility that the evolution of care-giving may have created novel selection pressures, selecting for immune responses that are adapted for a care-giving host. Such selection may underlie increases in immune system reactivity in humans relative to other great apes^67,68^ and may have contributed to the introgression of locally adapted immune genes as the human lineage colonized new habitats and hybridized with other hominin species^27–29^.

On a population scale, our results suggest that care-giving enabled kin-based cooperatively breeding hominins, with increasingly complex contact networks, to prevent disease transmission from increasing with social network complexity. The significance of care-giving as a mechanism of disease control is reinforced by research indicating that care-giving suppresses disease outbreaks more than an avoidance strategy^1^. During hominin evolution, care-giving likely produced a cascade of novel selection pressures, selecting for psychological changes that would facilitate cooperative care-giving, symptom advertisement for soliciting care, an immune system specialized for care-giving, and pathogens adapted to transmission among care-giving hosts. Care-giving was likely a key element of the psychological and behavioral traits that are associated with the success of the genus *Homo*^2–9^, and the ability to suppress disease spread may have been a prerequisite^21,23^ for the extreme social complexity that evolved in the human lineage.

## Methods

The world is a grid of 100 × 100 grid cells each of which represents 2 km^2^, making the community’s range 200 km^2^. This is within the confidence intervals of the calculated area required for *H*. *erectus*, *H*. *heidelbergensis*, *H*. *neandertalensis*, and *H*. *sapiens*^1,49^. Carrying capacities were set at 50, 100, 150, and 200 individuals to cover communities sizes observed in chimpanzees^51,52^, reconstructed values for hominins^1,49^, and values observed in some modern hunter-gather communities^53^. Reproduction occurs according to the formula:

1 $$(1-({\rm{number}}\,{\rm{of}}\,{\rm{agents}}/{\rm{carrying}}\,-\,{\rm{capacity}}))\ast {\rm{number}}\,{\rm{of}}\,{\rm{healthy}}\,{\rm{females}}$$

Females can have a maximum of one offspring per time step. Offspring are placed in a radius of one grid cell of the mother.

Each social system is reduced to its care-giving networks (Table S1), thus the networks are not synonymous with the mating system (see below for a discussion of the mating system in each social system). No attraction rules were used to subdivide the community into subgroups/kin groups or limit movements. At the end of each time step, healthy agents move randomly to an empty grid cell in a radius of 10. If no grid cells are available, the agent stayed. This produces a single, fission-fusion community in which subgroups of individuals are close enough to infect each other and other subgroups are not. The care-giving networks are a result each social system’s input rules for forming bonds between individuals (i.e., kin networks expand as individuals reproduce). Across social systems, networks differ in size and in modularity (the degree to which they are connected to other networks, details for each social system are provided below).

Each model initializes with 10 males and 10 females distributed randomly around the landscape. Each agent starts as uninfected and its personal disease parameters are set to 0: my-shedding (pathogen load), my-probability-transmission, my-probability-fatality, and my-probability-recovery. Each hominin is randomly assigned one of the four possible care-giving skills (providing food, water, hygiene assistance, or protection) and randomly assigned an intelligence score. Intelligence is conceptualized as a general ability for efficient information processing^54^, with more specific cognitive abilities like disease recognition and social learning being aspects of intelligence. These links between disease recognition, intelligence, and the processing of social information are supported by neurological research demonstrating that disease recognition involves integrating information from the odor and face perception networks^16^. This indicates that the brain pathways involved in disease recognition overlap with those which process social information from faces and odors^16^. Moreover, disease recognition does *not* require that individuals have an abstract concept of disease. Instead disease recognition functions on the ultimate level of causation, similarly to how kin recognition operates without individuals requiring an abstract concept of kin. In our models the hominin intelligence scores range from 0 to 1, thus scaling to the human IQ scores which range from 50 to 150 (Table 1). When an agent reproduces, the offspring’s intelligence is drawn from a normal distribution with the mother’s intelligence as the mean and a standard deviation of 0.15 (see below for social system specific differences). This represents the 15% standard deviation in the human IQ scale (Table 1).

The disease starts when the community reaches ½ of the carrying capacity, enabling the community to reproduce and establish its social network. When the disease begins, a percentage of the population (5%, 25%, 50%, or 75%) is randomly infected. This enabled us to test the effects of initial prevalence on the outcomes of the model. This approach is also logical and relevant to the real world. Depending upon sources and transmission modes, outbreaks vary greatly^69^. For example, a small number of individuals may have contact with another infectious individual, leading to very low initial prevalences in the population (i.e., tuberculosis transmission, known to be pre-agricultural^24^). Alternatively, if all community members drink from a contaminated water source (i.e., Salmonella transmission, known to be pre-agricultural^24^), initial prevalence may be very high. We expect that the evolution of care-giving strategies would have had to be able to cope with both low and high prevalence outbreaks.

We chose to create a spatially explicity model to enable the diseases to transmit through a community spread across a landscape. This represents the spatial component of disease transmission through a population. During an outbreak of a socially transmitted disease, individuals who are physically near infected individuals are more vulnerable than more distant individuals. This produces waves of infections and transmission along social networks^22,70–73^. By including a spatial dimension, we demonstrate that our model can cope with these complex dynamics.

We did not connect our population to an external reservoir because the aim of this study was to examine whether care-giving could control highly variable transmission dynamics. If we had connected our population to an external reservoir, it would have constrained the disease transmission dynamics, making it easier for the population to evolve effective care-giving mechanisms. Transmission dynamics can be extremely complex, involving multiple and changing host species, and multiple and changing transmission modes^69^. Instead of restricting the model, we varied the initial prevalence values across runs while holding the other parameters constant (disease parameters, community size).

When one disease goes extinct, the next starts in the following time step. Each disease has a death rate, transmission rate, and recovery rate. Infected agents set their pathogen load to 1000 (the absolute value is not important, it is a relative value), and set their personal probabilities of transmission, death, and recovery to those of the disease, and set the list of the types of care that they need to be food, water, hygiene assistance, and protection. When an agent is diseased, each agent in its care-giving network undergoes two screening processes, first based on disease recognition and second based on care-giving skills. (Care-giving networks are social system specific, details provided below). The potential carers each draw a random number from 0 to 1. If the number is below the agent’s intelligence score, it correctly identifies the diseased agent as needing care. If the number is above, the agent incorrectly identifies the diseased agent as healthy and will not provide care. Agents that correctly identify the disease are then screened according to whether they have one of the care-giving skills that the agent needs. Care types can only be given once per infection and each carer can only give one type of care to a given agent per time step. If the sick agent remains sick over multiple time steps, the selection process is repeated in each time step. This means that the same carer could provide a different form of care in a subsequent time step if it has more than one care-giving skill or learns another skill. Additionally, because intelligence is a probability of correctly identifying disease in others, carers that previously failed to recognize the disease may do so in subsequent time steps if the sick agent remains sick. If this occurs and these remaining agents have care-giving skills that the sick agent needs, they can provide care.

The remaining carers give care based on a modified version of Hamilton’s Rule^55^. Hamilton’s rule states that individuals should behave altruistically when: (benefit to the recipient) * (relatedness with the recipient) >(cost). We expressed the costs and benefits in terms of disease risk, such that:

2 $$\begin{array}{c}({\rm{benefits}}\,{\rm{to}}\,{\rm{the}}\,{\rm{recipient}})\ast ({\rm{relatedness}}\,{\rm{between}}\,{\rm{carer}}\,{\rm{and}}\,{\rm{diseased}}\,{\rm{agent}}) > ({\rm{death}}\,{\rm{rate}})\ast ({\rm{transmission}}\,{\rm{rate}}/{\rm{number}}\,{\rm{carers}}){\rm{.}}\end{array}$$

The benefits are based on medical literature (Table 1) documenting reductions in disease parameters (i.e., transmission, fatality). Benefits are calculated as the sum of the change in the agent’s disease parameters (probabilities of transmission, fatality, and recovery). In Hamilton’s rule, we divided the transmission rate by the number of carers (up to four, one for each care type) to reflect that carers providing cooperative care will be able to reduce the time spent in close proximity to the diseased individual, thus enabling each individual carer to minimize their exposure. Each type of care improves one of the agent’s personal disease parameters: probability of death, probability of transmission, probability of recovery. The new disease parameter is used to solve the exponential equation of pathogen replication for a reduced pathogen load and corresponding reductions in the other disease parameters. This also provides indirect benefits to future carers by lowering the probability of transmission. All carers, except for pair-partners and altruists, cease providing care when the costs exceed the benefits, according to a modified version of Hamilton’s rule^55^ in which the costs and benefits are expressed in terms of disease risk (infectiousness of the infected individual, death rate of the disease, and recovery rate).

After providing care, carers receive a random number. If it is below the probability of transmission/number of care types the agent received, then the carer is infected. If the carer is not infected, it attempts to learn new care-giving skills. Carers can learn skills from carers with whom they provided cooperative care. The carer goes through the list of types of care that the diseased agent received and draws a random number for each. If it is below the carer’s intelligence, then the carer adds that skill to its repertoire.

Diseased agents then receive a random number, if it is below their personal probability of death, then they die. If it is above, they survive to attempt recovery. They receive a random number and if it is below (1 − personal probability of recovery), then the agent recovers. If not, the agent remains diseased. Agents within 10 grid cells of a diseased agent select a random number. If it is below that diseased agent’s probability of transmission, the agent is infected. Healthy agents move to an unoccupied grid cell within a radius of 10 grid cells. Both the infection radius and movement radius are set to the same value (20 km^2^). This is the maximum distance which hunter-gatherers typically forage in a day^49,53^, thus it represents individuals close enough to interact with the diseased agent when it is infectious.

### Social systems

We reduced each of the social systems to its care-giving networks (Table S1). These networks are not synonymous with the mating system. We describe the care-giving networks and mating networks in each social system:

#### Basal primate

In most animal species, including primates^6^, the only extensive care-giving that occurs is from the mother to the offspring. This bond is given a relatedness value of 0.5, representing matrilineal relatedness. No cooperative care occurs, thus diseased individuals receive a maximum of one type of care. Females reproduce without forming bonds with males.

#### Pair-bonded

Pair-bonding has been argued to be the underlying structure of the human social systems, enabling both matrilineal and patrilineal kin recognition and facilitating cooperation across kin communities (via in-laws)^42^. While this may have been important for reducing violence and competition between communities^42^, pair-bonding and bisexual dispersal are associated with lower levels of kinship within communities^43,74^. Thus, in our model, the care-giving bonds are the basal mother-offspring bond, the father-offspring bond, and the pair-bond between the mother and father. Parent-offspring bonds receive relatedness values of 0.5, reflecting the genetic relationship. No grandparent or sibling care is given. Pair-partners provide obligate care to each other (if they recognize the disease and have the necessary care-giving skills), reflecting mating effort and/or reproductive investment in the mate. Unpaired males and unpaired females are randomly paired. To eliminate confounding effects of assortative mating by intelligence, pair-bonded males are assigned the intelligence scores of the female partner. This creates a simplified two parent system without introducing selection for an intelligent mate. Only pair-bonded females reproduce.

#### Indirect reciprocity

This social system reflects the literature in which meat sharing by hunting males in a community serves to bridge periods of ill health^44,45^. Males share food with individuals who are sick and unable to provide for themselves, and in the future when they, themselves, are ill, they receive food from community members. This reciprocity is indirect because the future givers may not be the same individuals who receive in the present^44,45^. We parameterize this social system to be the same as the pair-bonded system, except that diseased individuals receive food provisions from randomly selected male altruists. Food is provided regardless of the risks and regardless of disease recognition. As in the pair-bonded system, unpaired males and unpaired females are randomly paired. Only pair-bonded females reproduce.

#### Kin-based

This social system includes the basal mother-offspring care and care from matrilineal relatives with relatedness values of 0.125 or greater. This is consistent with the levels of kin recognition observed in nonhuman animals (Table 1). This includes sibling care^47^, grandparent care^14^, and care from other matrilineal relatives. It reflects the ethnographic literature on the BaYaka detailing how knowledge of medicinal plants is shared within, but not widely beyond, kin networks^59^. Care is bi-directional reflecting that care-giving occurs across generations, including contributions by immatures who would be capable of provisioning ill individuals with water, providing hygiene assistance, or providing some protection by staying with an ill individual. *Although this social system is conceptualized with females remaining in their natal communities*, *the findings are not specific to matrilineal kin networks*. In societies where females disperse^74^, patrilineal kin could provide care. Such kin-based care is not dependent upon continued investment by the father and would also be successful in promiscuous or polygynous societies^74^. As in the basal system, females reproduce without forming bonds with males.

### Two models

The Ineffective Care Model simulates the difficulties of establishing care-giving in a community that provides ineffective care while undergoing repeated introductions of novel diseases. Each time an agent provided care, the value of the benefits of the care was a random number between 0 and the maximum value found in the literature (Table 1). Because the disease parameters determine how the disease progresses through the community, novel diseases were created by assigning disease parameters random numbers between 0 and 0.5. We focused this disease range because, based on prior work^1^, that is where we expected the most care-giving to occur in all social systems.

The Effective Care Model examines the effects of social system and disease parameters once effective care-giving has been established in the population. Under this model, the benefits of care were fixed at the values found in the literature (Table 1). Disease parameters were varied simultaneously between 0.1 and 0.9. For example a disease with a severity of 0.1 had a death rate of 0.1, transmission rate of 0.1, and recovery rate of 0.9.

Each model was run under each of the four social systems. Carrying capacity was run with settings of 50, 100, 150, and 200 individuals. The initial prevalence of the disease was run at settings of 5%, 25%, 50%, and 75%. Each parameter combination, for each social system, for each model, was run 100 times. Runs were 1000 time steps. Runs were stopped and discarded if the population could no longer reproduce (i.e., there were less than two individuals of each sex or there were no healthy agents left).

### Statistical analysis

At the end of each run, the model output: mean number of care events received by diseased agents per infection (*cooperative care*), mean percentage of diseased agents to receive at least one type of care (*percent care*), mean number of care-giving skills per agent (*skills*), the net intelligence change between the start of the first time step and the end of the last (*intelligence change*), mean number of time steps required for a disease to go extinct (*disease extinction*), and mean percentage of agents infected by other agents (*disease spread*). Each of these parameters (except for intelligence change) was calculated separately for each disease event, then averaged across disease events to produce an average value for that run. We used a model fitting, not hypothesis testing, approach to examine how the output variables varied between social systems, and what predictor variables (i.e., community size, initial prevalence, disease severity) explained the variation between runs in each social system. We ran linear mixed models in R^75^ using lme4^76^ including all possible combinations of interactions. For each model, we grouped the data by social system and allowed the main effects to vary by social system, i.e., a random intercept and slope model design was used. Thus, for both models, social system was a random variable and the predictor variables and interaction effects were fixed (Ineffective Care Model’s fixed variables: Community Size, Initial Prevalence, Community Size * Initial Prevalence; Effective Care Model’s fixed variables: Community Size, Initial Prevalence, Disease Severity, and all corresponding interactions). We estimate both the marginal and conditional R2 for each model^77^. The marginal R2 estimates the ability of the model’s fixed effects to explain variation, whereas the conditional R2 takes into account both the fixed and random components, i.e., random intercept and slopes for each social system. The difference between the two measures provides an estimate of how much variation can be explained by the differences between social systems. We calculated AIC scores and present the results of the models which best fit the data (Table 2). Comparisons among all possible models are presented in Table S2.

We confirm that all methods were carried out in accordance with relevant guidelines and regulations. Because this study used no live subjects it did not require informed consent or institutional licensing.

## Electronic supplementary material

Supplementary information

Effective Care Model

Ineffective Care Model

## Data Availability

Code for the Ineffective Care and Effective Care Models has been provided in the Supplementary Materials. The.nlogo files can be opened with a text editor or run in Netlogo using the BehaviorSpace tool. The Supplementary Materials also contain ODD (Overview, Design Concepts, Details) protocols describing how to use the models. Code for the linear mixed models is available upon request. The datasets, including a list of the random seeds used in the runs, are available upon request. By uploading the random seed data into the models, users can reproduce the exact runs that output the datasets in this paper.
