# Supplementary material for: Social Structure Facilitated the Evolution of Care-giving as a Strategy for Disease Control in the Human Lineage
Source: Sci Rep. 2018 Sep 27;8:13997. doi: 10.1038/s41598-018-31568-2 (PMC6160448; doi:10.1038/s41598-018-31568-2)
Supplement: Supplementary file 1 — Supplementary information [file 41598_2018_31568_MOESM1_ESM.docx]

Social Structure Facilitated the Evolution of Care-giving as a Strategy for Disease Control in the Human Lineage

**Authors**:

Sharon E. Kessler, Tyler R. Bonnell, Joanna M. Setchell, Colin A. Chapman

Supplementary Materials:

Supplementary Text: ODD Model Descriptions of Ineffective and Effective Care Models

Figures S1-S3

Tables S1-S2

**Supplementary Text**

**ODD Protocol for *Ineffective Care Model* in Netlogo**

**Purpose**

The purpose of this model is to test 1) how the leading, competing hypotheses of ancestral hominin social structure facilitate or hinder the evolution of care-giving for the diseased under conditions of continual infection with novel diseases and ineffective care-giving and 2) how selection on care-giving varies based on community size and the initial prevalence of the disease.

**Entities, state variables and scales**

This model consists of three entities: the landscape, agents moving on the landscape, and links between agents. The landscape is a 100 x 100 cell grid which wraps horizontally and vertically. The model space simulates individuals moving and interacting on a landscape. The grid cells do not have any variables of their own.

The following global variables can be user-adjusted via the interface or set by BehaviorSpace:

1. **Carrying-capacity**: maximum number of agents on the landscape
2. **Num-agents:** number of unrelated pairs of agents created at set-up
3. **Initial**-**prevalence:** the number of agents who are randomly infected at the start of a new disease

Agents have the following state variables:

1. **Disease?:** a true/false variable determining the agent’s disease status
2. **Intelligence**: the probability that an agent will correct identify the disease status of another agent (0-1)
3. **Sex:** 0 or 1, assigned randomly
4. **My**-**shedding:** pathogen load of the agent
5. **My**-**prob**-**fatality:** likelihood that the agent will die of the disease (0-1). This value is reduced when the agent receives care.
6. **My**-**prob**-**transmission:** likelihood that the agent will transmit the infection to another agent within the infection radius (0-1). This value is reduced when the agent receives care.
7. **My**-**prob**-**recovery:** likelihood that the agent will recovery
8. **My**-**needs:** a list of the types of care the agent needs (food, water, hygiene, protection)
9. **M**y-**skills:** a list of the types of care-giving an agent can provide (food, water, hygiene, protection)
10. **Num**-**skills:** number of skills an agent has
11. **Care**-**sick**-**turtle**-**got:** list of care types the agent has received in that time step
12. **My**-**available**-**carers:** list of agents who may provide care to the agent
13. **My**-**carers:** list of agents who provided care, some of which may contract the disease, some of which may not
14. **My**-**learners:** list of agents who have provided care and did not contract the disease
15. **Starting**-**transmission:** likelihood of transmission at the start of a care-giving procedure, used to calculate costs for agents that provide care.
16. **Count**-**carers:** number of carers who might provide care to an ill agent, used to estimate the costs for agents providing cooperative care, capped at 4 because there are 4 types of help
17. **Diff**-**care:** difference between the number of carers who might have provided care and those who did. Used to calculate actual costs for agents who did provide care.
18. **My**-**total**-**benefit:** sum of benefits an agent received in the time step
19. **My**-**mean**-**benefit**: mean benefit an agent received per help per time step
20. **Helped:** number of times an agent received care during a given time-step (0-4)

Links have one variable, r, which represents the bond between the two agents (0-1 for genetic relatives, 100 for pair-partners). Links can be directional or not-directional. Directional links exist when care is only given in one direction (i.e., parent to offspring, but not the reverse). Not-directional links exist when care can be given in both directions (i.e., between pair-partners). The bonds vary according to social system:

1. **Basal primate:** Directional link from mother to offspring, r value of 0.5, colored white.
2. **Pair-bonded:** Directional link from parents to offspring, r values of 0.5, colored white. Not-directional link between pair-partners, r values of 100, colored green (produces obligate care in Hamilton’s rule below).
3. **Indirect reciprocity:** Includes the relationships of the pair-bonded system. Altruists and recipients do not form long-term bonds.
4. **Kin-biased:** Not-directional links between mothers and offspring (r=0.5, colored white), matrilineal siblings and grandparent/grandchildren (r=0.25, colored red), matrilineal aunts/uncles, nieces/nephews, and cousins (r=0.125, colored blue).

Simulations last for 1000 time steps. Agents reproduce at the beginning of each time step, but because no maximum life span is set, the time steps do not translate directly into generations or years.

**Process overview and scheduling**

Each time step, the following sequences occur:

1. The model runs the *initialize* procedure and sets a list of global tracking variables to 0 or false [see submodel *initialize* for details].
2. The population repopulates at each time step when healthy agents reproduce. This procedure produces the social system. There are three possible social systems: basal primate [*populate-basal2*], pair-bonded [*populate-pair2*], and kin-based cooperative breeding [*populate-coop*]. The indirect reciprocity system uses *populate-pair2*. ***The relevant social system should be activated by removing the semi-colon in front of that line of the “Go” procedure (lines 218-220), and adding a semi-colon in front of the populate procedures which are to be inactivated.*** Semi-colons comment out the line of code behind them. [See submodels *populate-basal2, populate-pair2, and populate-coop* for details].
3. Each agent’s links are reduced to only those that are relevant for the given social system:
   1. *Basal primate:* Links with an r value of less than 0.5 are removed. ***User must comment in the Prune-links-basal procedure in line 222.***
   2. *Pair-bonded:* Links with an r value of less than 0.5 are removed. ***User must comment in the Prune-links-pair procedure in line 223.***
   3. *Reciprocity:* Links with an r value of less than 0.5 are removed. ***User must comment in the Prune-links-pair procedure in line 223.*** The Indirect Reciprocity social system uses the procedure *prune-links-pair2.*
   4. *Kin-based:* Links with an r value of less than 0.125 are removed. ***User must comment in the Prune-links-coop procedure in line 221.***
4. The model checks whether the total number of agents is greater than half of the carrying capacity and if all the agents are healthy. If so, a percentage of agents equal to the value of *initial-prevalence* are randomly infected with the disease. Infected agents set *disease?* to true, turn a darker shade of blue, set *my-shedding* to 1000, set *my-needs* to the four types of care, and set their personal probabilities of transmission (*my-prob-transmission),* fatality (*my-prob-fatality)*, and recovery (*my-prob-recovery)* to the respective values of the disease [see submodel *start-disease* for details].
5. The *count-time-to-extinction-start* procedure determines whether there are any agents with disease? = true and if so, adds 1 to the variable *extinction-counter.* This procedure tracks the number of time steps it takes for the disease to go extinct.
6. The *pre-calculate* procedure sets *total-disease* to the number of agents who are infected, *total-turtles* to the number of agents, and *first-TS-intel* to the mean intelligence of the agents. (Note: The program language refers to agents as “turtles,” thus the variables “total-turtles” is the total number of agents.)
7. The model assesses whether there are any diseased agents and if so, the diseased agents locate potential carers (procedure *find-carers)* and receive care (procedure *request-care2*). In the Indirect Reciprocity social system, diseased agents solicit provisioning (procedure *provision*) before they request care. See submodels *find-carers, provision,* and *request-care2* for details.
8. The procedure *sort-carers* determines which carers will be infected and which will remain healthy and thus have the opportunity to learn new care-giving skills. Each carer receives a random number greater than or equal to 0 and less than 1. If the number is lower than the probability of transmission of the agent who received care at the time the care was given / the number of times the diseased agent received care, then the carer is infected with the disease. If the random number is higher, then the agent goes on to a list of carers who have the opportunity to learn additional care-giving skills.
9. Each carer that was not infected attempts to learn other care-giving skills. The carer draws a random number for each type of care that the diseased agent (for whom the agent provided care) received. If the random number is below the carer’s intelligence score, then the carer adds that skill to its list of skills (*my-skills*). If the number is above, then the carer does not learn that skill. Any duplicates are removed from the carer’s skill list.
10. In the procedure *post-calculate* the model calculates the following tracking variables: *helped1, helped2, helped3, helped4, total-helped, skilled-turtles, mean-help-received, diff-care, my-mean-benefit,* and *benefit-counter.* See submodel *post-calculate* for details.
11. In the procedure *disease-mortality* each infected agent receives a random number that is greater than or equal to 0 and less than 1. If the number is below the agent’s personal probability of fatality (*my-prob-fatality*), the agent dies. If it is above, the agent attempts recovery. Agents that attempt recovery receive another random number greater than or equal to 0 and less than 1, if it is below (1 – *my-prob-recovery*), the agent recovers and sets *disease?, my-shedding, my-prob-transmission, my-prob-fatality, my-prob-recovery, my-needs* to 0, false or empty. It returns to the lighter shade of blue. If the random number is above (1-*my-prob-recovery),* the agent remains diseased.
12. Each infected agent attempts to infect all healthy agents within a radius of 10 grid cells. These agents each receive a random number that is greater than or equal to 0 and less than 1. If the number is below the diseased agent’s *my-prob-transmission* the healthy agent is infected and 1 is added to G2 (which tracks agent-to-agent infections). If the random number is above the diseased agent’s *my-prob-transmission*, the agent is not infected.
13. Healthy agents move to an empty grid cell in a radius of 10. If no grid cells are available, the agent stays.
14. The model calculates variables for output and outputs data. Some data is output every time step, some at the end of a disease event, and some at the end of the run [see submodel *final-calculations* for details].

**Design concepts**

*Emergence:* Over time, because higher intelligence individuals have more accurate disease recognition and are better at learning new care-giving skills from others, they provide more care and are more likely to learn new skills. This means that when care-giving produces fitness advantages, higher intelligence individuals reproduce more than lower intelligence individuals. Higher population intelligence emerges. When the costs are higher than the fitness advantages, lower intelligence individuals who provide less care reproduce more than higher intelligence individuals. Lower population intelligence emerges.

*Adaptive behavior:* Agents receive an intelligence value that is drawn from a normal distribution with the mother’s intelligence as the mean and a standard deviation of 0.15 (scales to human IQ measures ^75^). Agents do not adapt over their lifetimes.

*Objectives:* Agents’ objectives are to maximize their own fitness by either providing care to kin or limiting their exposure by refusing to care for kin. Agents decide based on a modification of Hamilton’s rule of inclusive fitness [see submodel *help*].

*Learning:* When multiple agents provide care to the same diseased agent in the same time step, they can learn new care-giving skills from the other carers. This represents social learning.

*Prediction:* Agents explicitly calculate the potential costs and benefits when deciding whether to give care or avoid ill kin based on Hamilton’s rule [see submodel *help*].

*Sensing:* Agents know their own disease status, the disease characteristics of their infected kin (probability of fatality, probability of transmission, and probability of recovery after care), and their relatedness to all other agents (link variable: r). The accuracy with which they sense the disease status of their kin is based on their intelligence score (which they do not sense). Agents do not sense when they make mistakes.

*Interaction:* Agents interact directly by infecting and providing care to others. They also interact indirectly because when they provide care to a sick individual who recovers, they reduce the danger of infection for all other agents within the infection radius of that individual and for other potential carers with whom they may also be related.

*Stochasticity:* Disease parameters are represented as likelihoods in order to incorporate the uncertainty of disease transmission, mortality, and recovery.

*Collectives:* Care-giving networks are collectives of agents. Who recognizes one another as from the same networks depends on the social system.

*Observation:* For model testing, variables are output which quantify disease transmission, change in intelligence, and care-giving behavior [see submodel *final-calculations* for details].

**Set-up**

A random seed is generated and saved. This number can be used to reproduce the run. The program generates 2 * *num-agents*, half of which are male, half of which are female. Agents are randomly placed on the grid. Each of these agents is randomly assigned an intelligence value that is less than or equal to 0 and less than 1. Each agent is randomly assigned a care-giving skill to *my-skills*, and *num-skills* is set to 1. All other agent variables are set to 0/false/empty set (see agent’s state variables above). The lists of possible types of care that can be given is set to food, water, hygiene, and protection. Additional variables are:

- **Disease**-**counter**: This variable counts the number of disease events in the run
- **Setup**-**intel**: Mean intelligence of agents created at set-up

*Sets variables which sum within disease events to 0 at start of run. These variables are used in future calculations:*

- **Sum-prob-transmit:** cumulative sum of the probability of transmission from all time steps within the disease event. (Note: this does not vary within a disease event because it uses the base rate of the disease, not the personal values of the individual agents.)
- **Sum-prob-fatality:** cumulative sum of the probability of fatality from all time steps within the disease event. (Note: this does not vary within a disease event because it uses the base rate of the disease, not the personal values of the individual agents.)
- **Sum-prob-recovery:** cumulative sum of the probability of recovery from all time steps within the disease event. (Note: this does not vary within a disease event because it uses the base rate of the disease, not the personal values of the individual agents.)
- **Sum-extinction-counter:** cumulative sum of the number of time steps it takes for each disease to go extinct
- **SUM-D-event-intelligence:** cumulative sum of the mean intelligence for each disease event
- **SUM-D-event-intel-diff:** cumulative sum of the mean intelligence difference per time step of each disease event
- **SUM-D-event-pop:** cumulative sum of the mean population size per time step of the disease events. (Note that carrying capacity is fixed.)
- **SUM-D-event-total-disease**: cumulative sum of the mean count of sick agents who received care per time step of the disease event
- **SUM-D-event-prev:** cumulative sum of the mean prevalence of sick agents per time step of the disease events. (Note: starting prevalence is fixed.)
- **SUM-D-event-total-helped:** cumulative sum of the mean count of sick agents who received care per time step of the disease event
- **SUM-D-event-perc-helped:** cumulative sum of the mean percentage of sick agents who received care per time step of the disease event
- **SUM-D-event-help-received:** cumulative sum of the mean number of care events that sick individuals received per time step
- **SUM-D-event-perc-helped1:** cumulative sum of the mean percentage of sick turtles who received one care event each time step of the disease event
- **SUM-D-event-perc-helped2:**  cumulative sum of the mean percentage of sick turtles who received two care events each time step of the disease event
- **SUM-D-event-perc-helped3:** cumulative sum of the mean percentage of sick turtles who received three care events each time step of the disease event
- **SUM-D-event-perc-helped4:** cumulative sum of the mean percentage of sick turtles who received four care events each time step of the disease event
- **SUM-D-event-skilled:** cumulative sum of the mean number of agents with all four care-giving skills per time step of the disease event
- **SUM-D-event-perc-skilled:** cumulative sum of the mean percentage of agents with all four care-giving skills per time step of the disease event
- **SUM-D-event-mean-num-skills:** cumulative sum of the mean number of skills agents have per time step of the disease event
- **SUM-D-event-G2:** cumulative sum of the count of agents infected by other agents per time step of the disease event
- **SUM-D-event-G2-prev:** cumulative sum of the percentage of agents infected by other agents per time step of the disease event
- **SUM-D-event-mean-diff-care:** cumulative sum of the mean difference in agents’ numbers of potential carers and number of times care was received, per time step of the disease event
- **SUM-D-event-mean-benefit:** cumulative sum of the mean value of the care each agent received per time step of the disease event

*Sets variables which will be averages (across disease events within a run) to 0 at the start of the run*

- **RUN-intelligence:** cumulative sum of the mean intelligence of agents -- averaged over time steps within a disease event, then summed across disease events within the run
- **RUN-intel-diff:** cumulative sum of the mean intelligence difference per time step -- averaged over time steps within a disease event, then summed across disease events within the run
- **RUN-pop:** cumulative sum of the mean population size -- averaged over time steps within a disease event, then summed across disease events within the run
- **RUN-total-disease:** cumulative sum of the mean count of the total number of diseased agents -- averaged over time steps within a disease event, then summed across disease events within the run
- **RUN-prev:** cumulative sum of the mean disease prevalence -- averaged over time steps within a disease event, then summed across disease events within the run
- **RUN-total-helped:** cumulative sum of the mean count of agents who received care -- averaged over time steps within a disease event, then summed across disease events within the run
- **RUN-perc-helped:** cumulative sum of the mean percentage of agents who received care -- averaged over time steps within a disease event, then summed across disease events within the run
- **RUN-help-received:** cumulative sum of the mean number of times sick agents received care -- averaged over time steps within a disease event, then summed across disease events within the run
- **RUN-perc-helped1:** cumulative sum of the mean percentage of agents who received one care event -- averaged over time steps within a disease event, then summed across disease events within the run
- **RUN-perc-helped2:** cumulative sum of the mean percentage of agents who received two care events -- averaged over time steps within a disease event, then summed across disease events within the run
- **RUN-perc-helped3:** cumulative sum of the mean percentage of agents who received three care events -- averaged over time steps within a disease event, then summed across disease events within the run
- **RUN-perc-helped4:** cumulative sum of the mean percentage of agents who received four care events -- averaged over time steps within a disease event, then summed across disease events within the run
- **RUN-skilled:** cumulative sum of the mean number of agents with all four care-giving skills -- averaged over time steps within a disease event, then summed across disease events within the run
- **RUN-perc-skilled:** cumulative sum of the mean percent of agents with all four care-giving skills -- averaged over time steps within a disease event, then summed across disease events within the run
- **RUN-mean-num-skills:** cumulative sum of the mean number of skills per agent -- averaged over time steps within a disease event, then summed across disease events within the run
- **RUN-G2:** cumulative sum of the mean number of agents infected by other agents -- averaged over time steps within a disease event, then summed across disease events within the run
- **RUN-G2-prev:** cumulative sum of the mean percentage of agents infected by other agents -- averaged over time steps within a disease event, then summed across disease events within the run
- **RUN-mean-diff-care:** cumulative sum of the mean difference between the count of potential care-givers and the number of care-giving events received -- averaged over time steps within a disease event, then summed across disease events within the run
- **RUN-mean-benefit:** cumulative sum of the mean value of the care received by agents who received care -- averaged over time steps with a disease event, then summed across disease events within the run
- **Benefit-counter:** count of the number of time steps within a disease event in which at least one agent received care
- **RUN-benefit-counter:** count of the number of disease events in which at least one agent received care

**Input**

The user does not need to input additional files. A file with the random seeds used to produce data set in this paper can be provided upon request.

The user must select a social system under which to run the model. There are three possible social systems: basal primate [*populate-basal2*], pair-bonded [*populate-pair2*], and kin-based cooperative breeding [*populate-coop*]. ***The relevant social system should be activated by removing the semi-colon in front of that line of the “Go” procedure (lines 218-220), and adding a semi-colon in front of the populate procedures which are to be inactivated. The relevant “prune-links” procedure must also be activated by removing the semicolon in front of that line and inserting semicolons in front of the prune-links procedures that are to be inactivated.*** The *populate-basal2* uses the *prune-links-basal* procedure (line 222); *populate-pair2* uses *prune-links-pair* (line 223); and *populate-coop* uses *prune-links-coop* (line 221). The indirect reciprocity system uses *populate-pair2*, *prune-links-pair,* ***and also the*** ***provision*** submodel (semi-colon must be removed from line 233).

**Submodels**

*Initialize:* The model ***initializes the time step*** by having a set of global tracking variables set to 0/false. In addition each agent sets several of its’ own tracking variables to zero. These variables are used later to calculate and store values which will be output at the end of each time step.

Global tracking variables:

*These variables store values for calculations or output that occur later in the time step.*

- **Total**-**helped:** total number of agents who receive care (help)
- **Total**-**disease:** total number of agents who are diseased
- **Total**-**turtles:** total number of agents (called turtles by Netlogo)
- **Disease**-**counter:** count of diseases introduced per run; IDs each disease
- **Sum**-**prob**-**transmit:** stores a value used in later calculations for output of average probability of transmission across the run
- **Sum**-**prob**-**fatality:** stores a value used in later calculations for output of average probability of fatality across the run
- **Sum**-**prob**-**recovery:** stores a value used in later calculations for output of average recovery of transmission across the run
- **Extinction**-**counter:** tallies the number of time-steps until a disease event is extinguished
- **Sum**-**extinction**-**counter:** stores a value used in later calculations for output of average time to disease extinction across the run
- **Start**-**D**-**event**-**intel:** average intelligence of agents at the start of a disease event
- **First**-**TS**-**intel:** average intelligence at start of time step
- **Sum**-**intel**-**diff:** stores a value used in later calculations for output of average change in intelligence across the run
- **Helped1:** number of agents who received help once in a given time step
- **Helped2:** number of agents who received help twice in a given time step
- **Helped3:** number of agents who received help 3 times in a given time step
- **Helped4:** number of agents who received help 4 times in a given time step
- **Skilled**-**turtles:** number of agents possessing all four care-giving skills
- **Mean**-**help**-**received:** average number of helping events per agent that received help in that time-step
- **G2:** total number of agents infected by diseased agents
- **Seed:** to output random seed used for each run, can be used to reproduce the run
- **Mean**-**benefit:** mean benefit of agents
- **Benefit**-**counter** number of time steps within a disease event in which at least one agent received care
- **RUN-benefit-counter:** count of the number of disease events in which at least one agent received care
- **Setup**-**intel:** average intelligence of agents first created in setup procedure

*These variables hold values which are summed cumulatively across time steps within a disease event. They are also set to 0. See set-up for definitions.*

- **SUM-D-event-intelligence**
- **SUM-D-event-intel-diff**
- **SUM-D-event-pop**
- **SUM-D-event-total-disease**
- **SUM-D-event-prev**
- **SUM-D-event-total-helped**
- **SUM-D-event-perc-helped**
- **SUM-D-event-help-received**
- **SUM-D-event-perc-helped1**
- **SUM-D-event-perc-helped2**
- **SUM-D-event-perc-helped3**
- **SUM-D-event-perc-helped4**
- **SUM-D-event-skilled**
- **SUM-D-event-perc-skilled**
- **SUM-D-event-mean-num-skills**
- **SUM-D-event-G2**
- **SUM-D-event-G2-prev**
- **SUM-D-event-mean-diff-care**
- **SUM-D-event-mean-benefit**

*These variables hold values for calculations which are output at the end of each run. See set-up procedure for definitions.*

- **RUN-intelligence**
- **RUN-intel-diff**
- **RUN-pop**
- **RUN-total-disease**
- **RUN-prev**
- **RUN-total-helped**
- **RUN-perc-helped**
- **RUN-help-received**
- **RUN-perc-helped1**
- **RUN-perc-helped2**
- **RUN-perc-helped3**
- **RUN-perc-helped4**
- **RUN-skilled**
- **RUN-perc-skilled**
- **RUN-mean-num-skills**
- **RUN-G2**
- **RUN-G2-prev**
- **RUN-mean-diff-care**
- **RUN-mean-benefit**

*Populate-basal:* The population grows at each time step of the model when healthy agents reproduce according to the formula: ((1 - (number of agents / carrying-capacity)) * number of healthy females). Reproduction occurs asexually in that intelligence is inherited through the mother. No father is defined, representing a society in which there are no long-term bonds between fathers and mates, or fathers and offspring. Such kin-based care is not dependent upon continued investment by the father and would also be successful in promiscuous or polygynous societies^74^. Offspring are placed within a radius of 1 grid cell away from the mother, producing a population that is spatially structured by kinship. Each offspring’s intelligence is drawn from a normal distribution with the mother’s intelligence as the mean and a standard deviation of 0.15^75^. Offspring sex is randomly assigned. Offspring are randomly assigned one care-giving skill, representing independent discovery of the skill. *Num-skills* is set to 1. All other agent variables are set to 0/false/empty set (see agent’s state variables above). Mother-offspring relationships receive relatedness value (r) of 0.5. All links are directional, with care being given from the mother to the offspring.

*Populate-pair:* ***This social system is also used in the Indirect Reciprocity social system.*** At the start of this procedure, unpaired males and unpaired females form pair-bonds. Only one male and one female can be pair-bonded, simulating a monogamous mating and social system. The population grows at each time step of the model when healthy agents reproduce according to the formula: ((1 - (number of agents / carrying-capacity)) * number of healthy, pair-bonded females). Reproduction occurs asexually in that intelligence is inherited through the mother. To eliminate confounding effects of assortative mating by intelligence, pair-bonded males are assigned the intelligence scores of the female partner. This creates a simplified two parent system without introducing selection for an intelligent mate. Offspring are placed within a radius of 1 grid cell away from the mother, producing a population that is spatially structured by kinship. Each offspring’s intelligence is drawn from a normal distribution with the mother’s intelligence as the mean and a standard deviation of 0.15^75^. Offspring sex is randomly assigned. Offspring are randomly assigned one care-giving skill, representing independent discovery of that skill. *Num-skills* is set to 1. All other agent variables are set to 0/false/empty set (see agent’s state variables above). Mother-offspring and father-offspring relationships receive relatedness values (r) of 0.5 and the links are directional, with care being given only from parents to offspring. Pair-partners form a non-directional pair-bond and, if they recognize that the partner is diseased, provide care regardless of the disease risks.

*Populate-coop:* The population grows at each time step of the model when healthy agents reproduce according to the formula: ((1 - (number of agents / carrying-capacity)) * number of healthy females). Reproduction occurs asexually in that intelligence is inherited through the mother. No father is defined, representing a society in which there are no long-term bonds between fathers and mates, or fathers and offspring. Such kin-based care is not dependent upon continued investment by the father and would also be successful in promiscuous or polygynous societies, e.g., ^74^. Offspring are placed within a radius of 1 grid cell away from the mother, producing a population that is spatially structured by kinship. Each offspring’s intelligence is drawn from a normal distribution with the mother’s intelligence as the mean and a standard deviation of 0.15^75^. Offspring sex is randomly assigned. Offspring are randomly assigned one care-giving skill, representing an independent discovery of that skill. *Num-skills* is set to 1. All other agent variables are set to 0/false/empty set (see agent’s state variables above). Matrilineal relatedness is tracked by links between agents with the links containing the relatedness value (r). Parent-offspring relationships receive relatedness values of 0.5 and offspring inherit the links of the parent but with ½ the relatedness value. All links are non-directional, meaning that care can be given in both directions throughout the lifetime of the agents (i.e., mother to offspring and offspring to mother). Patrilineal relatedness is not included in the model in this social system. *Although this social system is conceptualized with females remaining in their natal communities, the findings are not specific to matrilineal kin networks.* In societies where females disperse^74^, patrilineal kin could provide care.

*Start-disease:* The disease parameters, *prob*-*transmission*, *prob-fatality,* and *prob-recovery,* are independently and randomly selected to be less than or equal to 0 and less than 0.5. This limit was chosen to focus on milder diseases because, based on prior work^1^, that is where we most expected differences in selection for care-giving. The model then solves for R*-transmission, R-fatality*, and *R-recovery* using an exponential dose response curve ^50,51^: P(response) = 1-exp(-R * dose). For example, for *R-transmission,* P(response) is the probability of transmission, R is R-transmission, and dose is *my-shedding* which is arbitrarily set to 1000. The relative change in dose is important, not the absolute value. The same is done for *R-fatality* and *R-recovery.* The R-values are conceptualized as the likelihood of one pathogen causing the response (see definitions above), and do not change during the duration of the disease. Each time a new disease is started, 1 is added to *disease counter,* and the *prob-transmission, prob-fatality,* and *prob-recovery* are added to *sum-prob-transmit, sum-prob-fatality,* and *sum-prob-recovery,* respectively. The mean intelligence of the agents at the start of the disease is calculated and stored in the variable *start-D-event-intel.* The following variables are set to 0 (see above for definitions):

- **Extinction-counter**
- **SUM-D-event-intelligence**
- **SUM-D-event-intel-diff**
- **SUM-D-event-pop**
- **SUM-D-event-total-disease**
- **SUM-D-event-prev**
- **SUM-D-event-total-helped**
- **SUM-D-event-perc-helped**
- **SUM-D-event-help-received**
- **SUM-D-event-perc-helped1**
- **SUM-D-event-perc-helped2**
- **SUM-D-event-perc-helped3**
- **SUM-D-event-perc-helped4**
- **SUM-D-event-skilled**
- **SUM-D-event-perc-skilled**
- **SUM-D-event-mean-num-skills**
- **SUM-D-event-G2**
- **SUM-D-event-G2-prev**
- **SUM-D-event-mean-diff-care**
- **SUM-D-event-mean-benefit**
- **Benefit-counter**

*Find-carers:* Diseased agents create an agent set of the healthy agents with whom they have non-directional links or a link that goes from another agent to them (i.e., from a parent to the offspring). **Note that the links vary according to the social system, thus the network of carers also varies by social system.** These are the agents’ *available-carers. Available-carers* are then screened according to whether they recognize that the diseased agent is infected. Each available carer is assigned a random number that is less than or equal to 0 and less than 1. If the number is above the available carer’s intelligence score, the agent fails to recognize the disease and is removed from the set of *available*-*carers.*

*Request-care2:* Each diseased agent with at least one type of care in *my-needs* and at least one available carer, requests care. For each need (i.e., food, water, hygiene, protection), the agent requests care iteratively from each of its available carers until it either receives that type of care or runs out of carers, at which point, the agent repeats the process with the next need. In the Ineffective Care model, the benefits from the care vary across care-giving events. Each care-giving event receives a benefit value as follows [see Table S2 for citations]:

1. Benefit of receiving food is a random number less than 0.25
2. Benefit of receiving water is a random number less than 0.2
3. Benefit of receiving hygiene assistance is a random number less than 0.5
4. Benefit of receiving protection is a random number less than 0.2

These benefits apply to each disease parameter (my-prob-transmission, my-prob-fatality, and my-prob-recovery of the diseased agent), and are summed as follows: *(Benefit*my-prob-transmission) + (benefit*my-prob-fatality) + (benefit*my-prob-recovery)* = *Hamilton*-*benefit*. The *Hamilton*-*benefit* quantifies the change in each of these disease parameters and is used in Hamilton’s rule. Carers decide whether to give care based on a modified version of Hamilton’s rule of inclusive fitness ^49^. In order for care to be given the following must be true: the benefit * relatedness > transmission risk * risk of fatality, where the benefit is *Hamilton-benefit,* the relatedness is the r value of the link between the two agents (for pair-partners r is 100, creating obligate care), the transmission risk is (*my-prob-transmission* of the diseased agent / the count of carers up to a maximum of four), and the risk of fatality is *my-prob-fatality* of the diseased agent. The probability of transmission is divided by the number of carers reflecting that when more carers are providing care, each carer can reduce the amount of time they provide in close proximity to the diseased agent. The maximum number of carers is four because there are only four types of possible care.

After Hamilton’s rule is fulfilled, the carer only gives care if the type of care sought (the need), is in the carer’s skill set. If so, then the diseased agent’s personal disease parameter is reduced by the benefit as described in the literature (Table S2):

1. Food reduces the probability of fatality, calculated as *benefit*my-prob-fatality*
2. Water reduces the probability of fatality, calculated as *benefit*my-prob-fatality*
3. Hygiene assistance reduces the probability of transmission, calculated as *benefit*my-prob-transmission*
4. Protection reduces the probability of fatality, calculated as *benefit*my-prob-fatality*

The reduced disease parameter is inserted back into the equation as P(response): P(response) = 1-exp(-R * dose) ^50,51^. The equation is solved for dose (*my-shedding),* using the new P(response value) and the corresponding R value calculated when the disease was seeded into the community. The reduced *my-shedding* is then inserted back into the equations for the other disease parameters and used to solve for reduced rates in those disease parameters. Thus, a reduction in one disease parameter reflects an **underlying reduction in the pathogen load** (*my-shedding),* which translates into corresponding changes in each disease parameter. These calculations reflect the additive benefits in the variable *Hamilton-benefit.*

After receiving care, that carer is removed from the diseased agent’s list of carers, the type of care received is removed from the diseased agent’s list of needs (*my-needs)*, and is instead inserted into the list of care that agent got (*Care-sick-turtle-got).* If the diseased agent has other types of care it needs, it solicits those types of care in the same way. Agents change their color according to the number of care types they received: 1 care type = yellow, 2 care types = orange, 3 care types = green, 4 care types = white. Agents can only receive a certain type of care once per illness.

*Provision:* This procedure is only run in the Indirect Reciprocity social system. Diseased agents run this procedure before requesting care from their care-giving network (*request-care2).* Diseased agents that have “food” on their list of *my-needs* request care from a healthy male agent who has the care-giving skill of “food.” This agent is selected at random without regardless of kinship. This reflects the literature on male meat-sharing in hunter-gather communities, e. g., ^39,41^. The benefit of the food is set at a random number below 0.25. Hamilton’s rule is not applied and food is provided regardless of the costs to the provisioner. As in *request-care2,* the care reduces the diseased agent’s disease parameters [see *request-care2* for details]. After providing care, the provisioner goes through the same process of determining whether it will be infected or learn new care-giving skills.

*Post-calculate:* The following tracking variables are calculated as follows:

- **Helped1:** number of agents who received 1 type of care
- **Helped2:** number of agents who received 2 types of care
- **Helped3:** number of agents who received 3 types of care
- **Helped4:** number of agents who received 4 types of care
- **Total-helped:** *helped1+helped2+helped3+helped4*
- **Skilled-turtles:** number of agents with all four care-giving skills
- **Mean-help-received:** mean number of care events received by agents who received care
- **Diff-care:** difference between the number of carers (up to 4) and the number of care events received
- **My-mean-benefit:** mean benefit of care events that agent received
- **Benefit-counter:** If care was given, 1 is added to benefit-counter

*Final-calculations:* The following variables are calculated at the end of each time step:

- **End-TS-intel:** mean intelligence of agents
- **TS-intel-diff:** *end-TS-intel* - *first-TS-intel*
- **Prevalence:** *total-disease* / *total*-*turtles* * 100
- **Perc-skilled:** *skilled-turtles* / *total-turtles* * 100
- **Mean-num-skills:** mean of *num-skills* of the agents
- **Perc-helped:** *total-helped* / *total-disease* * 100
- **G2-prev:** G2 / *total-turtles* * 100
- **Perc-helped1:** *helped1* / *total-helped* * 100
- **Perc-helped2:** *helped2* / *total-helped* * 100
- **Perc-helped3:** *helped3* / *total-helped* * 100
- **Perc-helped4:** *helped4* / *total-helped* * 100
- **Mean-diff-care:** mean of *diff-care* of agents who received care or had carers who could have given care

At the end of time step 1, the run’s random seed, the experiment name in behavior space, the run number, the carrying capacity, and the initial prevalence of the disease are written to each of the following datasheets. Then the following variables are output each time step to the corresponding datasheets:

- Datasheet: Intelligence.txt, **Variable**: *End-TS-intel*
- Datasheet: Intel_diff.txt, **Variable**: *TS-intel-diff*
- Datasheet: Pop_size.txt, **Variable**: *Total-turtles*
- Datasheet: Total_disease.txt, **Variable**: *total-disease*
- Datasheet: Prevalence.txt, **Variable**: *prevalence*
- Datasheet: Total-helped.txt, **Variable**: *total-helped*
- Datasheet: Perc_helped.txt, **Variable**: *perc-helped*
- Datasheet: Mean_help_received.txt, **Variable**: *mean-help-received*
- Datasheet: Perc_helped1.txt, **Variable**: *Perc_helped1*
- Datasheet: Perc_helped2.txt, **Variable**: *Perc_helped2*
- Datasheet: Perc_helped3.txt, **Variable**: *Perc_helped3*
- Datasheet: Perc_helped4.txt, **Variable**: *Perc_helped4*
- Datasheet: Skilled.txt, **Variable**: *skilled-turtles*
- Datasheet: Perc_skilled.txt, **Variable**: *Perc-skilled*
- Datasheet: Mean_num_skills.txt, **Variable**: *mean-num-skills*
- Datasheet: G2.txt, **Variable**: *G2*
- Datasheet: Perc_G2.txt, **Variable**: *G2-prev*
- Datasheet: Mean_diff_care.txt, **Variable**: *mean-diff-care*
- Datasheet: mean_benefit.txt, **Variable**: *Mean-benefit*

Each time step ***within a given disease event***, the following cumulative sums are calculated:

- **SUM-D-event-intelligence**: *SUM-D-event-intelligence + end-TS-intel*
- **SUM-D-event-intel-diff**: *SUM-D-event-intel-diff + TS-intel-diff*
- **SUM-D-event-pop:** *SUM-D-event-pop + total-turtles*
- **SUM-D-event-total-disease:** *SUM-D-event-total-disease + total-disease*
- **SUM-D-event-prev:** *SUM-D-event-prev + prevalence*
- **SUM-D-event-total-helped:** *SUM-D-event-total-helped + total-helped*
- **SUM-D-event-perc-helped:** *SUM-D-event-perc-helped + perc-helped*
- **SUM-D-event-help-received:** *SUM-D-event-help-received + mean-help-received*
- **SUM-D-event-perc-helped1:** *SUM-D-event-perc-helped1 + perc-helped1*
- **SUM-D-event-perc-helped2:** *SUM-D-event-perc-helped2 + perc-helped2*
- **SUM-D-event-perc-helped3:** *SUM-D-event-perc-helped3 + perc-helped3*
- **SUM-D-event-perc-helped4:** *SUM-D-event-perc-helped4 + perc-helped4*
- **SUM-D-event-skilled:** *SUM-D-event-skilled + skilled-turtles*
- **SUM-D-event-perc-skilled:** *SUM-D-event-perc-skilled + perc-skilled*
- **SUM-D-event-mean-num-skills:** *SUM-D-event-mean-num-skills + mean-num-skills*
- **SUM-D-event-G2**: *SUM-D-event-G2 + G2*
- **SUM-D-event-G2-prev:** *SUM-D-event-G2-prev + G2-prev*
- **SUM-D-event-mean-diff-care**: *SUM-D-event-mean-diff-care + mean-diff-care*
- **SUM-D-event-mean-benefit**: *SUM-D-event-mean-benefit + mean-benefit*

The following calculations are made at the end of each disease event. They are the means per time step within the current disease event for each variable:

- **D-event-intelligence**: *SUM-D-event-intelligence / end-extinction-counter*
- **D-event-intel-diff** : *SUM-D-event-intel-diff / end-extinction-counter*
- **D-event-pop**: *SUM-D-event-pop / end-extinction-counter*
- **D-event-total-disease**: *SUM-D-event-total-disease / end-extinction-counter*
- **D-event-prev**: *SUM-D-event-prev / end-extinction-counter*
- **D-event-total-helped**: *SUM-D-event-total-helped / end-extinction-counter*
- **D-event-perc-helped**: *SUM-D-event-perc-helped / end-extinction-counter*
- **D-event-mean-help**-**received**: *SUM-D-event-help-received / end-extinction-counter*
- **D-event-perc-helped1**: *SUM-D-event-perc-helped1 / end-extinction-counter*
- **D-event-perc-helped2**: *SUM-D-event-perc-helped2 / end-extinction-counter*
- **D-event-perc-helped3**: *SUM-D-event-perc-helped3 / end-extinction-counter*
- **D-event-perc-helped4**: *SUM-D-event-perc-helped4 / end-extinction-counter*
- **D-event-skilled**: *SUM-D-event-skilled / end-extinction-counter*
- **D-event-perc-skilled**: *SUM-D-event-perc-skilled / end-extinction-counter*
- **D-event-mean-num-skills**: *SUM-D-event-mean-num-skills / end-extinction-counter*
- **D-event-G2**: *SUM-D-event-G2 / end-extinction-counter*
- **D-event-G2-prev**: *SUM-D-event-G2-prev / end-extinction-counter*
- **D-event-mean-diff-care**: *SUM-D-event-mean-diff-care / end-extinction-counter*
- **D-event-mean-benefit:** *SUM-D-event-mean-benefit / benefit-counter*

At the end of each disease event the following variables are output into a spreadsheet titled *Disease_event_summary.txt.* Definitions are provided here and above when the variables are first introduced.

- **Seed**: output of random seed, makes run repeatable and serves as run tracker across runs
- **BehaviorSpace**-experiment-name: experiment name
- **BehaviorSpace**-run-number: run number
- **Carrying**-**capacity**: Max. number of agents that can be on the landscape
- **Initial-prevalence**: Prevalence at which a new disease starts
- **Disease-counter**: sequential numbering of disease events
- **Prob-transmission**: probability of transmission of this disease (starting value for agents before care is given)
- **Prob-fatality**: probability of fatality of this disease (starting value for agents before care is given)
- **Prob-recovery**: probability of recovery of this disease (starting value for agents before care is given)
- **End-extinction-counter**: number of time steps until disease went extinct
- **D-event-intelligence**: mean intelligence of agents across time steps of the disease event
- **D-event-intel-diff**: mean change in intelligence per time step of the disease event
- **D-event-pop**: mean population size across time steps of the disease event
- **D-event-total-disease**: mean count of diseased agents per time step of the disease event
- **D-event-prev**: mean disease prevalence per time step of the disease event
- **D-event-total-helped**: mean number of sick agents who got help each time step of the disease event
- **D-event-perc-helped**: mean percent of sick agents who got help each time step of the disease event
- **D-event-mean-help-received**: mean number of helps sick individuals received per time step of the disease event
- **D-event-perc-helped1**: mean percent of sick agents who got 1 help each time step of the disease event
- **D-event-perc-helped2**: mean percent of sick agents who got 2 helps each time step of the disease event
- **D-event-perc-helped3**: mean percent of sick agents who got 3 helps each time step of the disease event
- **D-event-perc-helped4**: mean percent of sick agents who got 4 helps each time step of the disease event
- **D-event-skilled**: mean number of agents each times step of the disease event who have all four care-giving skills
- **D-event-perc-skilled**: mean percent of agents with all four care-giving skills each time step of the disease event
- **D-event-mean-num-skills**: mean number of skills of agents in each time step of the disease event
- **D-event-G2**: total agents infected by other agents each time step of the disease event
- **D-event-G2-prev**: prevalence of agents infected by other agents each time step of the disease event
- **D-event-mean-diff-care**: mean difference in the number of potential care-givers and actual number of care-giving received per time step of the disease event
- **D-event-mean-benefit**: mean benefit received by agents who were helped per time step of the disease event

The following variables are calculated. They sum the means per disease event *across disease events* within the run:

- **RUN-intelligence**: *RUN-intelligence + D-event-intelligence*
- **RUN-intel-diff**: *RUN-intel-diff + D-event-intel-diff*
- **RUN-pop**: *RUN-pop + D-event-pop*
- **RUN-total-disease**: *RUN-total-disease + D-event-total-disease*
- **RUN-prev**: *RUN-prev + D-event-prev*
- **RUN-total-helped**: *RUN-total-helped + D-event-total-helped*
- **RUN-perc-helped**: *RUN-perc-helped + D-event-perc-helped*
- **RUN-help-received**: *RUN-help-received + D-event-mean-help-received*
- **RUN-perc-helped1**: *RUN-perc-helped1 + D-event-perc-helped1*
- **RUN-perc-helped2**: *RUN-perc-helped2 + D-event-perc-helped2*
- **RUN-perc-helped3**: *RUN-perc-helped3 + D-event-perc-helped3*
- **RUN-perc-helped4**: *RUN-perc-helped4 + D-event-perc-helped4*
- **RUN-skilled**: *RUN-skilled + D-event-skilled*
- **RUN-perc-skilled**: *RUN-perc-skilled + D-event-perc-skilled*
- **RUN-mean-num-skills**: *RUN-mean-num-skills + D-event-mean-num-skills*
- **RUN-G2**: *RUN-G2 + D-event-G2*
- **RUN-G2-prev**: *RUN-G2-prev + D-event-G2-prev*
- **RUN-mean-diff-care**: *RUN-mean-diff-care + D-event-mean-diff-care*
- **RUN-mean-benefit**: *RUN-mean-benefit + D-event-mean-benefit*

The following variables/calculations are output into a file called “Run_summary.txt”. They summarize the data of the entire run:

- **Seed**: random seed of the run
- **BehaviorSpace**-experiment-name: Experiment name
- **BehaviorSpace**-run-number: Run number
- **Carrying**-**capacity**: Max. number of agents the landscape can hold
- **Initial**-**prevalence**: The prevalence at which a disease starts
- **Disease**-**counter**: Total number of disease events in the run
- **Mean probability of transmission** over the run: *sum-prob-transmit / disease-counter*
- **Mean probability of fatality** over the run: *sum-prob-fatality / disease-counter*
- **Mean probability of recovery** over the run: *sum-prob-recovery / disease-counter*
- **Mean number of time steps until disease extinction**: *sum-extinction-counter / disease-counter*
- **Net**-**intel**-**diff:** *end-TS-intel - setup-intel*
- **Mean intelligence** (averaged over time steps within a disease event, then across disease events within the run): *RUN-intelligence / disease-counter*
- **Mean intelligence difference** (averaged over time steps within a disease event, then across disease events within the run): *RUN-intel-diff / disease-counter*
- **Mean population size** (averaged over time steps within a disease event, then across disease events within the run): *RUN-pop / disease-counter*
- **Mean total diseased agents** (averaged over time steps within a disease event, then across disease events within the run): *RUN-total-disease / disease-counter*
- **Mean disease prevalence** (averaged over time steps within a disease event, then across disease events within the run): *RUN-prev / disease-counter*
- **Mean** **number** **of** **agents** **who** **received** **care** (averaged over time steps within a disease event, then across disease events within the run): *RUN-total-helped / disease-counter*
- **Mean** **percentage** **of** **agents** **who** **received** **care** (averaged over time steps within a disease event, then across disease events within the run): *RUN-perc-helped / disease-counter*
- **Mean number of times sick agents got care** (averaged over time steps within a disease event, then across disease events within the run): *RUN-help-received / disease-counter*
- **Mean percentage of helped agents who received one type of care** (averaged over time steps within a disease event, then across disease events within the run): *RUN-perc-helped1 / disease-counter*
- **Mean percentage of helped agents who received two types of care** (averaged over time steps within a disease event, then across disease events within the run): *RUN-perc-helped2 / disease-counter*
- **Mean percentage of helped agents who received three types of care** (averaged over time steps within a disease event, then across disease events within the run): *RUN-perc-helped3 / disease-counter*
- **Mean percentage of helped agents who received four types of care** (averaged over time steps within a disease event, then across disease events within the run): *RUN-perc-helped4 / disease-counter*
- **Mean number of agents with all four care-giving skills** (averaged over time steps within a disease event, then across disease events within the run): *RUN-skilled / disease-counter*
- **Mean percentage of agents with all four care-giving skills** (averaged over time steps within a disease event, then across disease events within the run): *RUN-perc-skilled / disease-counter*
- **Mean number of skills per agent** (averaged over time steps within a disease event, then across disease events within the run): *RUN-mean-num-skills / disease-counter*
- **Mean number of agents infected by other agents** (averaged over time steps within a disease event, then across disease events within the run): *RUN-G2 / disease-counter*
- **Mean percentage of agents infected by other agents** (averaged over time steps within a disease event, then across disease events within the run): *RUN-G2-prev / disease-counter*
- **Mean difference between the number of potential care-givers and the number of care-giving events received** (averaged over time steps within a disease event, then across disease events within the run): *RUN-mean-diff-care / disease-counter*
- **Mean benefit** received by agents who got care (averaged over time steps with a disease event, then across disease events within the run): *RUN-mean-benefit / RUN-benefit-counter*
- **final**-**skilled**: net difference between number of agents with all four care-giving skills at the start and end of the run

**Model implementation**

The model is implemented in Netlogo 6.0.1 and can be run using the buttons on the interface or through the BehaviorSpace tool.

If run through the interface buttons, the model will run once and continue beyond 1000 time steps.

If run in BehaviorSpace, enter 1 for the number of runs to be conducted in parallel (BehaviorSpace/Run Options window). This will prevent data from data from multiple runs being intermixed in the output files. The model will run 100 runs at each parameter combination (community size: 50, 100, 150, 200 and initial prevalence: 5%, 25%, 50%, and 75%) for a total of 1600 runs. ***The model must be run separately for each social system.***

**ODD Protocol for *Effective Care Model* in Netlogo**

**Purpose**

The purpose of this model is to test 1) how the leading, competing hypotheses of ancestral hominin social structure facilitate or hinder the evolution of care-giving for the diseased under conditions of effective care-giving and 2) how selection on care-giving varies based on disease severity, community size, and the initial prevalence of the disease.

**Entities, state variables and scales**

Same as Ineffective Care Model with one additional variable which can be set in the user interface or in BehaviorSpace:

The following global variables are set in BehaviorSpace:

1. **Disease-parameter:** This variable is used to set *prob-transmission, prob-fatality,* and *prob-recovery* to a single value for an entire run. The values vary from 0.1 to 0.9 in increments of 0.1.

**Process overview and scheduling**

The process overview and scheduling is the same as in the Ineffective Care Model, except for the following changes:

1. *Start-disease:* Disease parameters *prob-transmission, prob-fatality,* and *prob-recovery* are set to *disease-parameter* which is set either by the user or BehaviorSpace. Values range from 0.1 to 0.9 in increments of 0.1. Disease severity (parameter: *Disease-parameter)* does not vary across disease events as it does in the Ineffective Care Model, but instead is constant throughout all the disease events in the run. Thus disease severity is varied systematically across runs, facilitating teasing apart the effects of disease severity on care-giving behavior and intelligence changes.
2. *Request-care2:* The benefits of each type of care are fixed at values documented in the medical literature [see Table S2 for citations]. The benefits are fixed both within and across runs, simulating a community that is able to consistently provide effective care.

- Benefit of receiving food is a 25% reduction in the probability of fatality
- Benefit of receiving water is a 20% reduction in the probability of fatality
- Benefit of receiving hygiene assistance is a 50% reduction in the probability of transmission
- Benefit of receiving protection is a 20% reduction in the probability of fatality

1. *Provision:* As in the Ineffective Care Model, *provision* is only used in the Indirect Reciprocity social system.

- Benefit of receiving food is a 25% reduction in the probability of fatality

1. *Final-calculation:* In time step one when the model prepares the data files for the data which will be output at the end of each time step, it outputs the following data for the run. Note that disease severity (parameter: *disease-parameter*) is output three times reflecting that it is the probability of transmission, probability of fatality, and probability of recovery.

- Seed
- BehaviorSpace Experiment Name
- BehaviorSpace Run Number
- *Disease-parameter*: probability of transmission (starting value for agents before care is given)
- *Disease*-*parameter*: probability of fatality (starting value for agents before care is given)
- *Disease*-*parameter*: probability of recovery (starting value for agents before care is given)
- *Carrying*-*capacity*
- *Initial*-*prevalence*

**Design concepts**

*Emergence:* Same as Ineffective Care Model

*Adaptive behavior:* Same as Ineffective Care Model

*Objectives:* Same as Ineffective Care Model

*Learning:* Same as Ineffective Care Model

*Prediction:* Same as Ineffective Care Model

*Sensing:* Same as Ineffective Care Model

*Interaction:* Same as Ineffective Care Model

*Stochasticity:* The Effective Care Model has less stochasticity than the Ineffective Care model because the probabilities of transmission, fatality, and recovery are fixed across disease events within a run. However, these probabilities are still represented as likelihoods in order to incorporate the uncertainty of disease transmission, mortality, and recovery. In addition the benefits of each type of care (food, water, hygiene, and protection) are fixed both within and across runs.

*Collectives:* Same as Ineffective Care Model

*Observation:* Same as Ineffective Care Model

**Set-up**

The same as the Ineffective Care Model, except that the disease characteristics (*prob-transmission*, *prob-fatality,* and *prob-recovery*) are varied systematically across runs at values of 0.1-0.9 at increments of 0.1.

**Input**

Same as Ineffective Care Model. Note that the user must still select a social system.

**Submodels**

Same as Ineffective Care Model, with changes described above in Process Overview and Scheduling

**Model implementation**

Same as Ineffective Care Model, except that the model will run 100 runs at each parameter combination (community size: 50, 100, 150, 200, initial prevalence: 5%, 25%, 50%, and 75%, and disease severity: 0.1, 0.2, 0.3, 0.4, 0.5, 0.6, 0.7, 0.8, 0.9) for a total of 14400 runs. The model must be run separately for each social system.

**Supplementary Tables**

**Table S1.** Care giving networks in each social system. Kin selection indicates that care is given when the fitness benefits of providing care are greater than the costs to the carer, as expressed by disease risks.

| **Relationship** | **Conditions** | **Basal Primate** | **Pair-bonded** | **Indirect Reciprocity** | **Kin-based** |
| --- | --- | --- | --- | --- | --- |
| Mother-Offspring | Kin selection | Mother to Offspring | Mother to Offspring | Mother to Offspring | Bi-directional |
| Father-Offspring | Kin selection | No | Father to Offspring | Father to Offspring | Bi-directional |
| Between Pair-partners | Obligate | No | Bi-directional | Bi-directional | no |
| Altruist Care | Obligate | No | No | Altruist to Recipient | no |
| Sibling Care | Kin selection | No | No | No | Bi-directional |
| Grandparent Care | Kin selection | No | No | No | Bi-directional |
| Other Kin Care | Kin selection | No | No | No | Bi-directional |

**Table S2.** Model comparisons between the full model with no interactions and full models with all possible combinations of interactions. AIC scores are subtracted, so that the best model is zero and all other are presented as a distance from the best model. CS is community size, DS is disease severity, and IP is initial prevalence.

| **Ineffective care models** | **No interactions** | **CS*IP (All interactions)** | | |  |
| --- | --- | --- | --- | --- | --- |
| Δ Intelligence | **0** | 12 | | |  |
| Cooperative Care | **0** | 12 | | |  |
| Skills | 140 | **0** | | |  |
| Spread | 200 | **0** | | |  |
| Percent Care | 92 | **0** | | |  |
| Extinction | 5 | **0** | | |  |
| **Effective care models** | **No interactions** | **CS*DS** | **DS*IP** | **CS*IP** | **All interactions** |
| Δ Intelligence | 199 | 192 | 14 | 164 | **0** |
| Cooperative Care | 2581 | 2253 | 554 | 2587 | **0** |
| Skills | 1001 | 1013 | 177 | 802 | **0** |
| Spread | 9017 | 7101 | 7394 | 4908 | **0** |
| Percent Care | 1538 | 1002 | 966 | 1122 | **0** |
| Extinction | 2894 | 1158 | 1936 | 2719 | **0** |

**
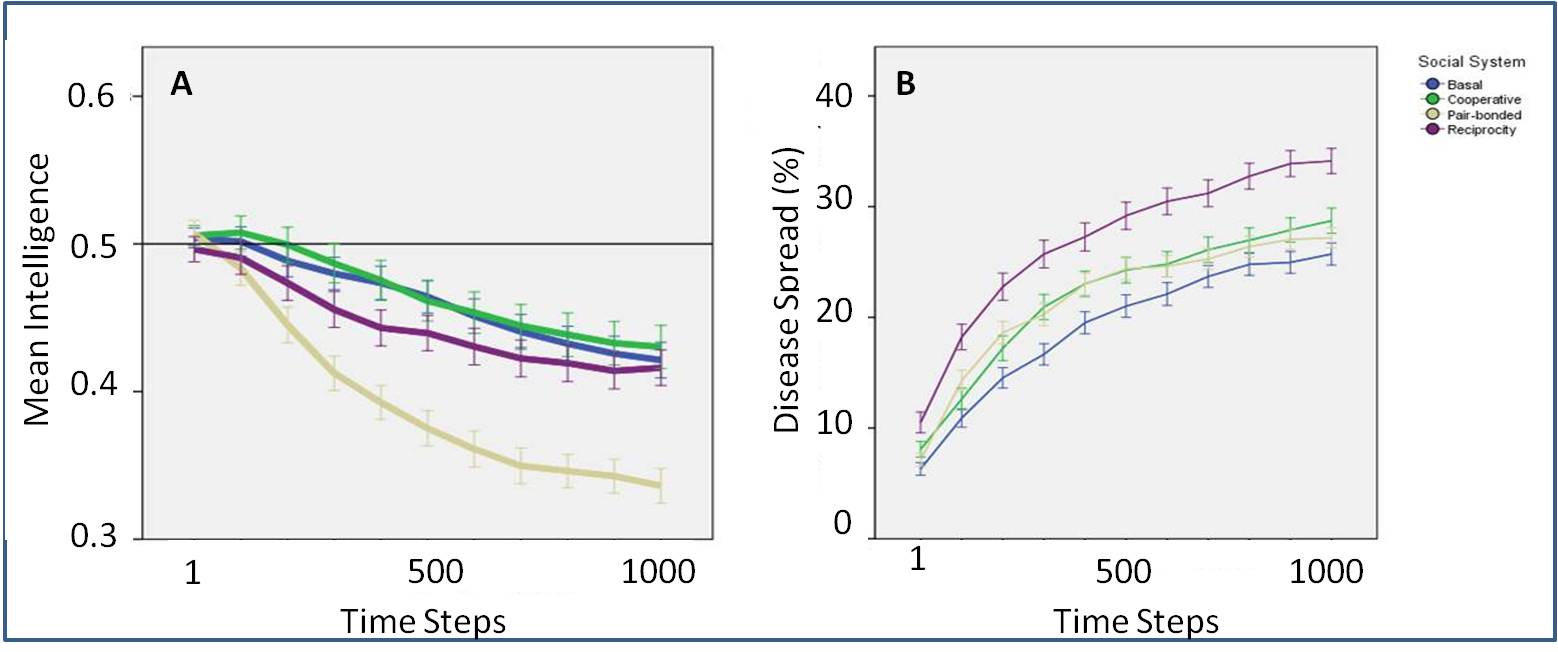
**

**Figure S1.** Change over time in the large communities’ mean population intelligence (A) and disease spread (B). The reference line at 0.5 (A) highlights the average intelligence of the populations at the start of the model runs. In the model, disease recognition is a function of intelligence, thus an increase in intelligence reflects selection for disease recognition and care-giving. The basal primate, pair-bonded, indirect reciprocity, and kin-based social systems are shown in blue, grey, purple, and green, respectively. The error bars are 95% confidence intervals.

**
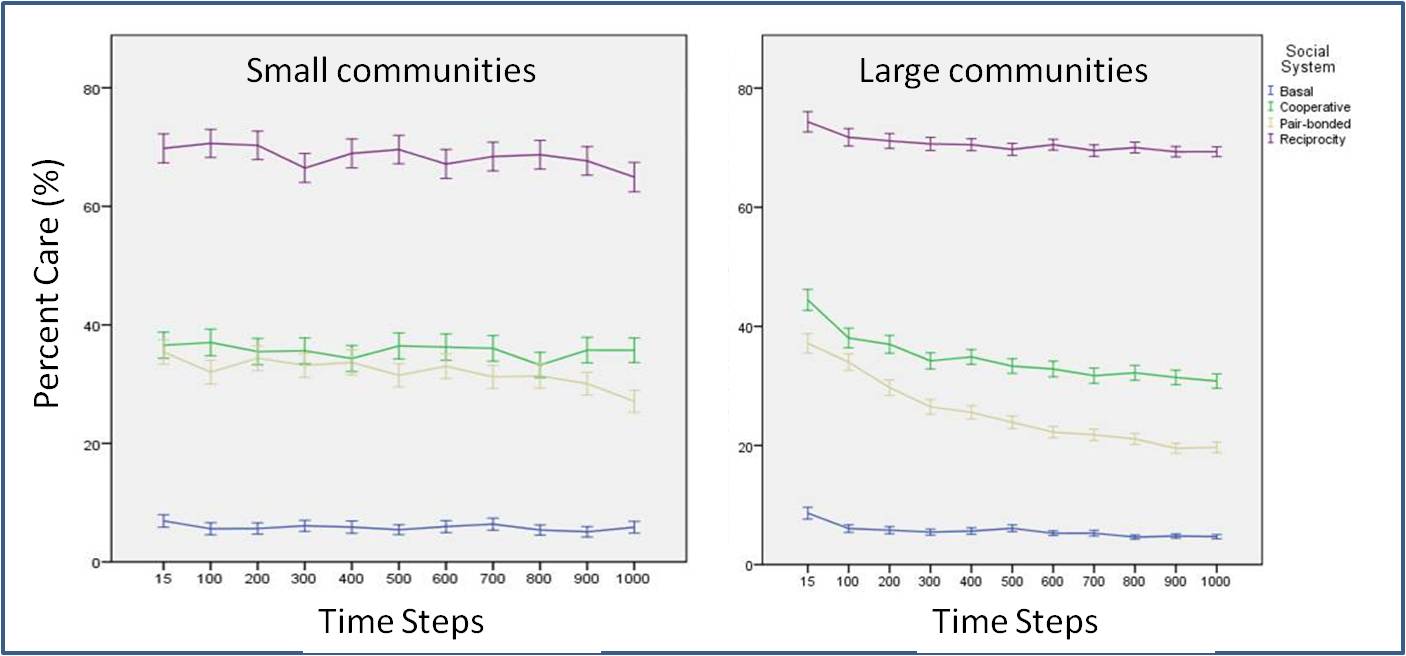
**

**Figure S2.** Percentage of diseased individuals in each social system to receive at least one type of care under the Ineffective Care Model. The basal primate, pair-bonded, indirect reciprocity, and kin-based social systems are shown in blue, grey, purple, and green, respectively. The error bars are 95% confidence intervals.

**
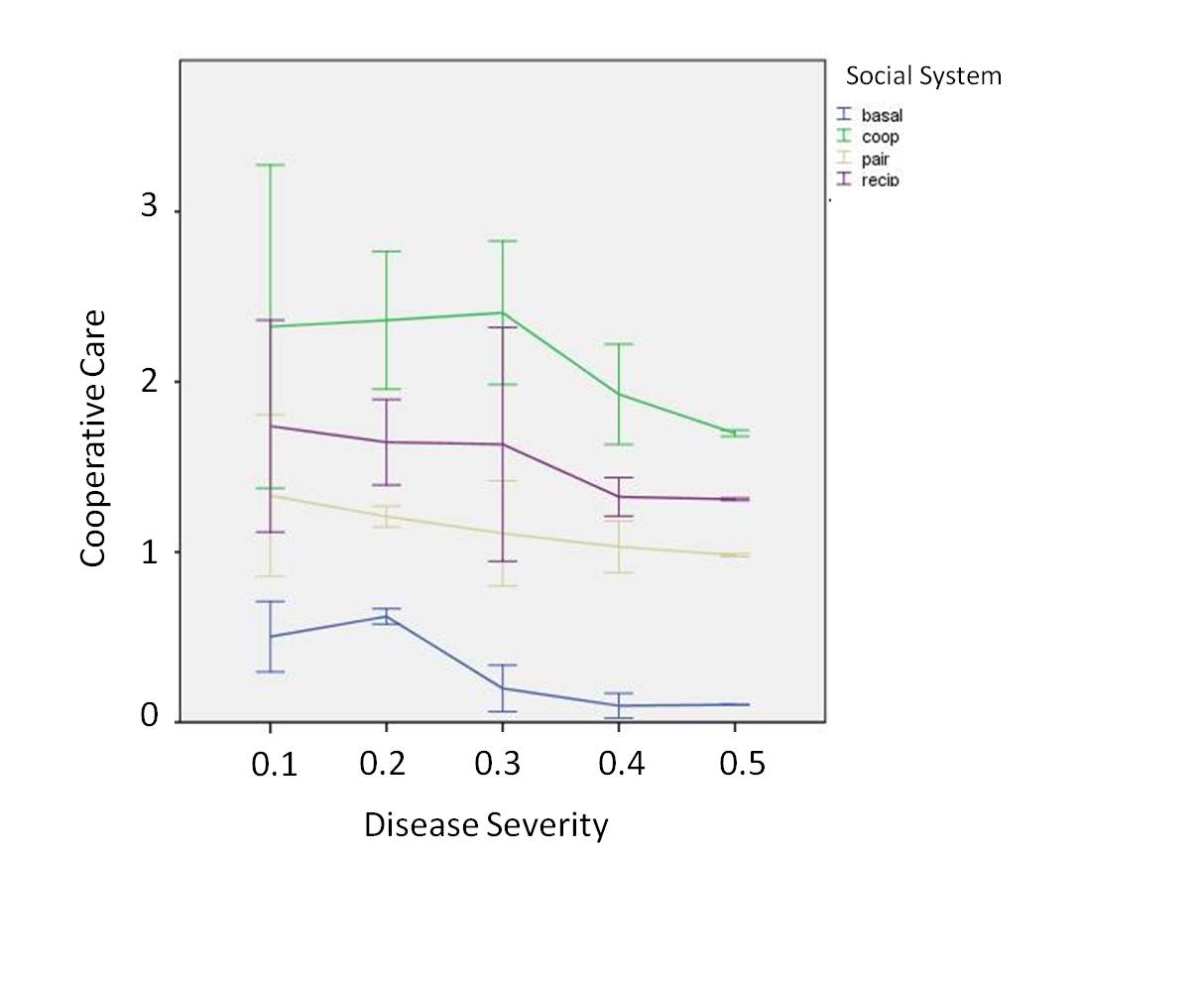
**

**Figure S3.** Levels of cooperative care produced by each social system under the Effective Care Model. Each diseased individual could receive up to four types of care. The kin-based social system produced the highest levels of cooperative care. The basal system did not produce cooperative care (multiple care events per diseased recipient). The graph pools across community sizes and initial disease prevalence settings. The basal primate, pair-bonded, indirect reciprocity, and kin-based social systems are shown in blue, grey, purple, and green, respectively. The error bars are 95% confidence intervals.
